# Multiscale topology classifies cells in subcellular spatial transcriptomics

**DOI:** 10.1038/s41586-024-07563-1

**Published:** 2024-06-19

**Authors:** Katherine Benjamin, Aneesha Bhandari, Jessica D. Kepple, Rui Qi, Zhouchun Shang, Yanan Xing, Yanru An, Nannan Zhang, Yong Hou, Tanya L. Crockford, Oliver McCallion, Fadi Issa, Joanna Hester, Ulrike Tillmann, Heather A. Harrington, Katherine R. Bull

**Affiliations:** 1 Mathematical Institute, University of Oxford, Oxford, UK; 2 Centre for Human Genetics, University of Oxford, Oxford, UK; 3 Nuffield Department of Medicine, University of Oxford, Oxford, UK; 4 Chinese Academy of Medical Sciences Oxford Institute, University of Oxford, Oxford, UK; 5 BGI Research, Riga, Latvia; 6 College of Life Sciences, University of Chinese Academy of Sciences, Beijing, China; 7 BGI Research, Qingdao, China; 8 Translational Research Immunology Group, Nuffield Department of Surgical Sciences, University of Oxford, Oxford, UK; 9 Isaac Newton Institute for Mathematical Sciences, University of Cambridge, Cambridge, UK; 10 Max Planck Institute of Molecular Cell Biology and Genetics, Dresden, Germany; 11 Centre for Systems Biology, Dresden, Dresden, Germany; 12 Faculty of Mathematics, Technische Universität Dresden, Dresden, Germany

**Keywords:** Transcriptomics, Computational biology and bioinformatics, Applied mathematics

## Abstract

Spatial transcriptomics measures in situ gene expression at millions of locations within a tissue^1^, hitherto with some trade-off between transcriptome depth, spatial resolution and sample size^2^. Although integration of image-based segmentation has enabled impactful work in this context, it is limited by imaging quality and tissue heterogeneity. By contrast, recent array-based technologies offer the ability to measure the entire transcriptome at subcellular resolution across large samples^3–6^. Presently, there exist no approaches for cell type identification that directly leverage this information to annotate individual cells. Here we propose a multiscale approach to automatically classify cell types at this subcellular level, using both transcriptomic information and spatial context. We showcase this on both targeted and whole-transcriptome spatial platforms, improving cell classification and morphology for human kidney tissue and pinpointing individual sparsely distributed renal mouse immune cells without reliance on image data. By integrating these predictions into a topological pipeline based on multiparameter persistent homology^7–9^, we identify cell spatial relationships characteristic of a mouse model of lupus nephritis, which we validate experimentally by immunofluorescence. The proposed framework readily generalizes to new platforms, providing a comprehensive pipeline bridging different levels of biological organization from genes through to tissues.

## Abstract (web summary)

A method for topological automatic cell type classification across subcellular resolution spatial transcriptomic platforms is proposed, resolving cell type information and locating sparsely dispersed cells in human kidney and mouse kidney and brain.

## Main

An open problem in spatial transcriptomics is the inference of information at the level of single cells^1^. Although recent experimental technologies enable whole-transcriptome measurement of gene expression at subcellular spatial resolutions, new computational methods are still required to infer single-cell information. Here we provide mathematical tools to fill this gap without previous knowledge of cell boundaries.

**Fig. 1:**
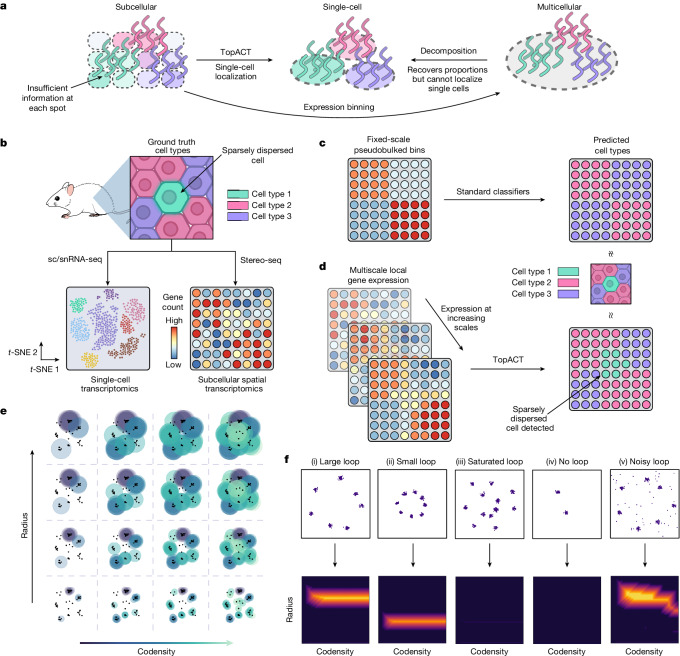
Method overview. **a**, Spatial transcriptomics measurements can be grouped according to their relative scale: subcellular, single-cell and multicellular. Existing methods decompose multicellular readings into cell type proportions. With subcellular data, it is necessary to aggregate data to reach the single-cell level. **b**, Automatic spatial cell type identification requires integration of single-cell and spatial transcriptomics. **c**, A common approach is to aggregate expression into a fixed-window and then run a standard classifier; however, this fails to resolve individual cells and can lose information about sparsely dispersed cells caught between bin boundaries. **d**, TopACT minimizes information loss by taking a flexible topological approach. Each spot is classified independently using local neighbourhoods at several scales, accommodating heterogeneous cell sizes and varying per-spot transcriptional abundance. This flexibility allows detection of finer structural information, including individual sparsely dispersed cells. **e**, A radius–codensity bifiltration defined on a two-dimensional point cloud. At each radius–codensity pair, a ball of that radius is drawn on top of all points with at most the given codensity (that is, sparseness). The hue indicates the underlying point codensity. The radius parameter therefore changes the scale of interaction between points and the codensity parameter controls the level of noise reduction. **f**, Archetypal spot assignment patterns (top) and their corresponding MPH landscapes (bottom). (i) The large loop structure activates the landscape at high radius values. (ii) The small loop structure activates the landscape at low radius values. (iii) A saturated loop structure with central clusters does not activate the landscape. (iv) A point cloud with no underlying loop structure does not activate the landscape. (v) The codensity parameter ensures that the landscape is still activated even in the presence of outliers and misclassifications. Panels **a**–**d** were created with BioRender (https://BioRender.com).

Spatial transcriptomics experiments have until now featured a trade-off between spatial resolution, transcriptome depth and sample size^2^. A wide range of methods^2,10^ have been developed to automatically predict cell types from both multicellular-resolution array-based data^11–13^ (Fig. 1a, ‘Decomposition’) and subcellular-resolution imaging-based data^14–18^. Most approaches focus on integration with single-cell or single-nucleus RNA sequencing (scRNA-seq or snRNA-seq, respectively) (Fig. 1b). Methods for multicellular data can broadly be separated into two categories: imputation methods, such as Seurat^19^, Tangram^20^ or CellTrek^21^, and decomposition methods, typically based on non-negative matrix factorization^12,22^ or statistical models^23–27^. Most methods for subcellular imaging-based data rely on upstream cell segmentation followed by standard cell type clustering^2^. Recent alternatives, such as Baysor^28^, assign cell types directly to transcripts without a segmentation step; however, this approach requires individual spatial localization of each transcript.

The inception of subcellular-resolution array-based technologies such as high-definition spatial transcriptomics (HDST) (about 2 μm)^3^, Visium HD (about 2 μm)^4^, Seq-Scope (about 0.5–0.8 μm)^5^ and Stereo-seq (0.5–0.715 μm)^6^ necessitates the development of bespoke mathematical methods for cell type identification. As high spatial resolution is associated with low per-spot transcriptional abundance, the opposite approach to decomposition is required: to recover single-cell information from subcellular data, reads from neighbouring spots must be aggregated to the single-cell level. When cell boundaries are unknown, a ‘fixed-window’ approach is often taken, for which a traditional decomposition method is used on a coarsely binned grid^5,6^ (Fig. 1a, ‘Expression binning’). However, by its nature this approach discards the advantages of the high-resolution platform^2^, rendering the inference of single-cell information impossible. Moreover, the fixed grid size and offset lead to underdetection of sparsely dispersed cells, such as immune cells, which are of critical importance in clinical settings (Fig. 1c).

Mathematically, the challenge of cell segmentation in this setting is to aggregate gene expression information across scales without previous knowledge of cell boundaries. To tackle this problem, we propose a method for topological automatic cell type identification (TopACT) (Fig. 1d), which independently classifies the cell type of each spot using a dynamically scaled local neighbourhood. Once cell types have been identified, we further develop this multiscale approach using multiparameter persistent homology^7–9^ (MPH), an emerging tool in the field of topological data analysis, which enables quantification and comparison of the spatial distributions of individually resolved cells across several samples (Fig. 1e,f).

Here, we demonstrate the ability of TopACT to produce spot-level cell type annotations with high accuracy, recovering single-cell-level structure that is inaccessible from fixed-window approaches, across a range of platforms and tissues. We showcase the method on mouse lupus kidney data generated by Stereo-seq, in which MPH analysis generates a hypothesis on glomerular immune cell organization which is confirmed with multiplex immunofluorescence imaging.

## TopACT

TopACT provides spot-level cell type annotations of subcellular spatial transcriptomics data. We assume that the aggregation of expression vectors from enough spots of the same cell type yields an expression vector comparable to an sc/snRNA-seq expression vector of that cell type. We then use this assumption to approach spatial cell type classification as a transfer learning problem, leveraging the high efficacy of automatic cell type classification in the single-cell setting^29^. By default, TopACT learns from an annotated sc/snRNA-seq reference sample to construct a single-cell classifier. This classifier is applied at several scales of aggregation, producing a multiscale cell type confidence matrix at each spot. By analysing this matrix, TopACT outputs a predicted cell type for each spot, from which individual cells can be resolved (Supplementary Fig. 2 and Methods).

## MPH landscapes

We use MPH^8^ to quantify the spatial organization of cell types produced by TopACT. MPH uses a multiparameter collection of lenses to filter through data by different metrics (Fig. 1e) and summarizes the overall geometry in a feature vector called an MPH landscape^9^. In the MPH landscape, different activation patterns correspond to different topological structures, allowing for the inference of qualitative structural information (Fig. 1f and Methods).

## Overview of results

We demonstrate TopACT on four datasets. We first benchmark the proposed method against the fixed-window decomposition approach on a synthetic Voronoi model. Next, we demonstrate the ability to discriminate a macrophage population not detectable by existing approaches in previously published Stereo-seq mouse brain data^6^. We then improve on the cellular morphology and cell annotation in a Xenium^17^ imaging-based spatial transcriptomic human kidney dataset. Finally, we showcase TopACT on a mouse kidney model by pinpointing the location of sparsely dispersed immune cells in new Stereo-seq data. We demonstrate a significant increase in glomerular immune activity in treated samples, consistent with lupus-like immune infiltration. MPH then quantifies the spatial patterning of immune cells in treated kidney samples, predicting a ring structure in treated glomerular immune cells. This purely spatial transcriptomic prediction is verified with multiplex immunofluorescence imaging for T cell, B cell and myeloid markers showing increased glomerular immune staining in treated kidney, with a peripheral distribution pattern.

## Benchmarking TopACT using synthetic data

**Fig. 2:**
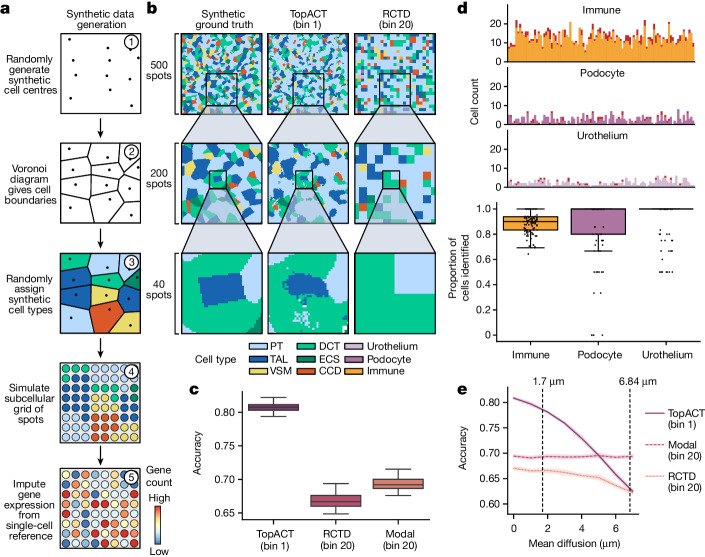
Benchmarking TopACT with synthetic data. **a**, Synthetic data generation schematic. **b**, Sample output of cell type identification algorithms on synthetic data. From left to right: ground truth, TopACT, RCTD. Colours indicate cell types. **c**, Box plots of per-iteration accuracy of cell type classification methods on synthetic data (*n* = 100 iterations). Centre line shows median; box limits show interquartile range (IQR); whiskers show full range. Modal is the optimal bin 20 classification assigning to each bin its most common ground truth cell type. **d**, TopACT performance on rare cell types. Top, number of cells of each type detected per iteration. Coloured regions denote cells detected by TopACT, red regions denote cells not detected by TopACT. Bottom, box plots showing recall of sparsely dispersed cells (*n* = 100 iterations). Centre lines show median; box limits show interquartile range; whiskers show full range of non-outlier points. Outliers are points more than 1.5× IQR from the upper or lower quartiles. Full distributions overlaid. **e**, Accuracy of methods under simulated molecular diffusion. Methods run for *n* = 10 iterations each on mean diffusion magnitudes of *λ* μm for *λ* = 0,1,2,…,7. Lines show mean, bands show s.e.m. Vertical dashed lines refer to previous diffusion estimates in literature^8,25^. CCD, cortical collecting duct; DCT, distal convoluted tubule; ECS, endothelial cells; PT, proximal tubule; TAL, thick ascending limb of the loop of Henle; VSM, vascular smooth muscle. Source Data

We use TopACT to classify cell types in synthetic benchmark data (Fig. 2a). The method is able to resolve fine structural detail, including sparsely dispersed cells (Fig. 2b). For comparison we run robust cell type decomposition (RCTD)^24^, an established decomposition method, using the standard fixed-window approach. We benchmark the accuracy of these methods and find that the mean accuracy for TopACT (*M* = 0.808, s.d. = 0.006) surpasses that of both RCTD (*M* = 0.668, s.d. = 0.010) and the theoretically optimal fixed-window classification (*M* = 0.693, s.d. = 0.009) achieved by assigning each bin its modal ground truth cell type (Fig. 2c).

To probe the ability of our method to identify sparsely dispersed cells, we produce predicted cell loci from the spot-level TopACT predictions for three rare cell types: Immune, Podocyte and Urothelium. This allows us to determine the proportion of ground truth cells that have an associated TopACT-predicted cell (defined as having centre-to-centre distance of, at most, 20 spots). Across 100 iterations, we find that TopACT consistently identifies all but a few of the cells of each rare cell type and often identifies all of them, validating the ability of the method to pinpoint rare cells (Fig. 2d).

Subcellular discrimination with array-based spatial transcriptomics may be limited by RNA diffusion. Estimates for RNA diffusion vary by platform and measurement, from a mean lateral diffusion distance of 1.7 μm using Seq-Scope, based on comparison between haematoxylin and eosin (H&E) stain and complementary DNA fluorescent labelling^11^, to 6.84 μm with Stereo-seq, based on the distance of vasoactive intestinal peptide (*Vip*) transcripts in interneurons from the cell centroid^6^. The latter of these figures is likely to be an overestimate, as not all *Vip* RNA will be located at the predicted cell centroid. To investigate the effect of diffusion on model performance, we test the performance of TopACT and RCTD under diffusion effects of increasing magnitude and find that the performance of our method remains robust under a range of levels of diffusion (Fig. 2e). In particular, TopACT continues to outperform the theoretically optimal bin 20 classification up to an average diffusion of 5 μm.

## Localizing macrophages in mouse brain

**Fig. 3:**
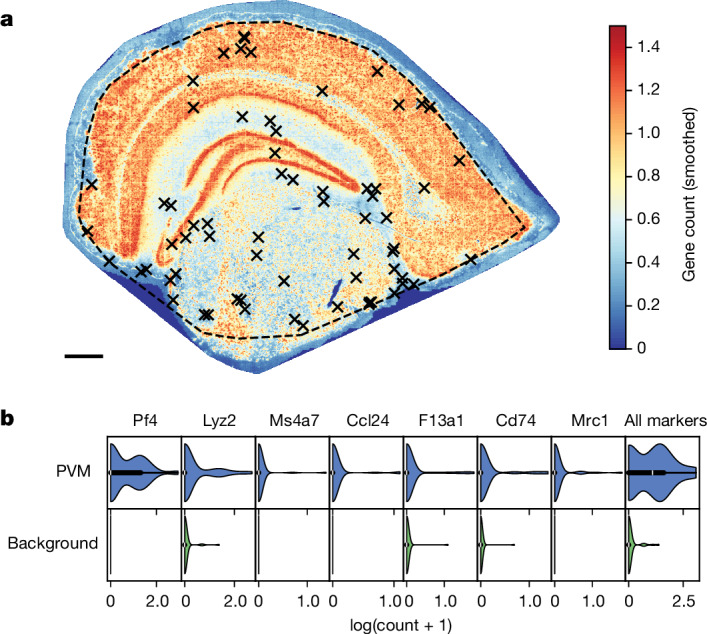
TopACT predicts previously unidentified PVM cells in adult mouse brain. **a**, Spatial locations of TopACT-predicted PVM cells (black crosses). Background heatmap shows smoothed transcript count. Black dashed line shows convex hull of high-density regions, to which analysis is restricted. Scale bar, 0.5 mm. **b**, Violin plots for expression of common markers of PVM cells, for TopACT-predicted PVM cells (blue, *n* = 66 cells) and randomly sampled background cells (green, *n* = 66 cells), across the entire mouse brain sample. Each plot corresponds to the expression counts of a single given marker gene in cells labelled with the given cell type. Violins show kernel density estimate of data distribution. Inner box-and-whisker shows summary statistics as follows: white centre line shows median; box limits show IQR; whiskers show full range. A log scale is used. Source Data

We first set out to examine the ability of TopACT to detect small and sparsely distributed cells against existing data. Published analysis of mouse brain data generated with Stereo-seq^6^ considered 50 × 50 nanoball bins as well as separately using a cell segmentation based on a nucleic acid stain. However, both of these approaches failed to detect any immune populations other than microglia, behaviour which was attributed to issues with cell segmentation. Here, we use TopACT to pinpoint perivascular macrophage (PVM) cells in these data, recovering an immune subpopulation which evaded detection with standard methods and validating the ability of the method to identify rare and sparely dispersed cell populations (Fig. 3a). We validate these findings by confirming high expression of PVM marker genes in TopACT-predicted PVM cells (Fig. 3b).

## Extending TopACT to imaging platforms

**Fig. 4:**
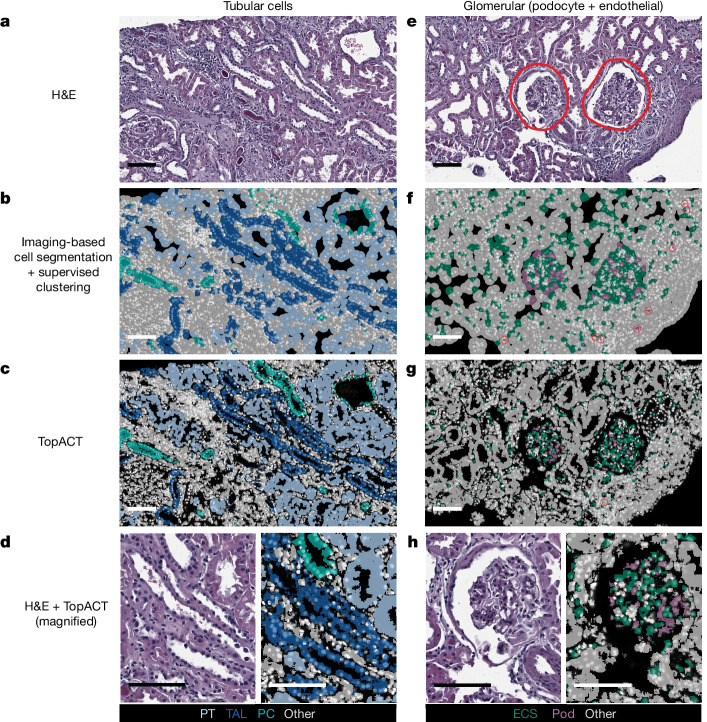
TopACT cell segmentation of human IgAN kidney profiled on the Xenium platform. **a**, H&E staining of an exemplar tissue region including tubular structures. **b**, Supervised tubular cell type annotation on imaging-based cell segmentation. Cell types profiled are proximal tubule (pale blue), thick ascending limb (dark blue) and principal cell (PC, turquoise). **c**, TopACT tubular cell type annotations. **d**, Comparison of H&E (left) and TopACT-annotated (right) tubular region at higher magnification. **e**, H&E staining of an exemplar tissue region showing glomeruli, marked with red circles. **f**, Supervised podocyte (Pod, purple) and endothelial (green) cell type annotation on imaging-based cell segmentation. Cells annotated as podocytes outside glomerular regions are identified by red circles. **g**, TopACT podocyte and endothelial cell type annotations. A single pixel (1 μm) outside the glomerular regions, marked with a red circle, is identified as containing podocyte markers but does not meet size criteria to be defined as a cell. **h**, Comparison of H&E (left) and TopACT-annotated (right) glomerular region at higher magnification. All annotations overlaid on DAPI fluorescent imaging showing nuclei distribution. All images are representative regions of a single Xenium experiment. Scale bars, 100 μm.

**Extended Data Fig. 1:**
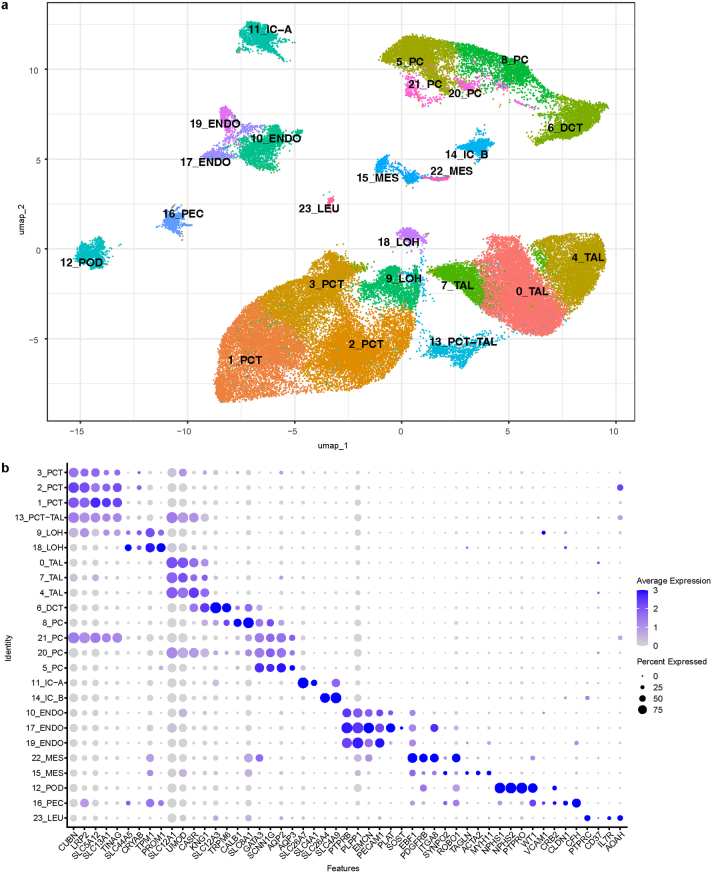
Reference snRNA-seq data from four healthy human kidney biopsies. **a**, UMAP embedding with labelled cell clusters. **b**, Dot plot showing expression of key marker genes for each cell type. PCT = proximal convoluted tubule; TAL = Thick ascending limb; LOH = loop of Henle; DCT = distal convoluted tubule; PC = principal cell; IC-A = α intercalated cell, IC-B = β intercalated cell; ENDO = endothelial cell; MES = mesangial cell; POD = podocyte; PEC = parietal epithelial cell; LEU = Leucocyte.

In situ RNA profiling technologies with increasingly large gene panels offer high capture efficiency to spatially profile genes and pathways of interest. These systems largely rely on imaging-morphology-based cell segmentation. To establish whether TopACT can be applied to these platforms and effectively annotate cells when given fewer genes, we generated a human kidney dataset, from an IgA nephropathy (IgAN) patient biopsy section, on the Xenium platform (Fig. 4). IgAN is the most common cause of glomerulonephritis and is due to deposition of complexes of abnormally glycosylated immunoglobulin A in the renal glomerulus^30^. We applied a standard panel of 377 genes not specifically developed for kidney^31^. An integrated snRNA-seq kidney dataset from healthy kidney donors was used for both TopACT and conventional Seurat supervised cell annotation (Extended Data Fig. 1).

In comparison to H&E staining of the same section (Fig. 4a), the standard imaging-based segmentation produces cells that lack granular structure and heterogeneity of shape, as demonstrated for tubule segments across the nephron, namely proximal tubule, thick ascending limb of the loop of Henle and distal tubular principal cells (Fig. 4b). By contrast, the morphology of the TopACT-predicted renal tubule segments, including the lumen space, are more biologically plausible and consistent with H&E staining of the same section (Fig. 4c,d).

Podocytes are highly specialized visceral epithelial cells which are exclusively found in renal glomeruli (Fig. 4e) as part of the filtration barrier. Conventional supervised clustering of imaging-based cell segments identifies a population labelled as podocytes localized to both glomerular and extra-glomerular regions (Fig. 4f). TopACT identifies a population of podocytes correctly located entirely in glomeruli (Fig. 4g,h).

These Xenium findings indicate that TopACT can accurately identify cell types and locations from targeted imaging-based spatial data and that integrating transcriptional information into segmentation can improve on current image-dependent methods.

## Pinpointing immune cells in mouse kidney

Inflammation is a key driver in several kidney diseases, including glomerulonephritis and diabetic nephropathy but typically, in these disorders, immune cells do not aggregate or form large lymphoid structures within the kidney. Spatially resolving sparsely dispersed immune cells in kidneys is challenging; immune cells may be small and are metabolically inactive compared to abundant tubular cells and therefore, in a fixed bin containing several cell types, tubular cell signatures will dominate.

**Fig. 5:**
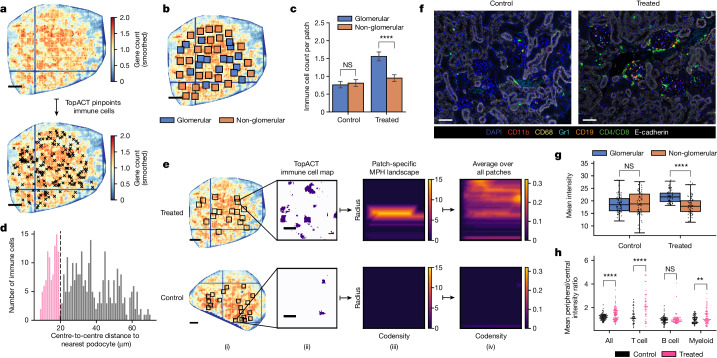
TopACT predicts immune cell ring structure in mouse glomeruli. **a**–**e**, Analysis of Stereo-seq kidney sections (four control, six treated). **a**, Example TopACT-predicted immune cells. Background, transcript density. Scale bars, 0.2 mm. **b**, Example glomerular (blue) and non-glomerular (orange) patch distribution. Scale bar, 0.2 mm. **c**, Mean TopACT-predicted immune count per patch, by condition and patch type. Error bars, s.e.m. Increased immune cell numbers (*P* = 4.2 × 10^−5^) observed in glomerular (*n* = 161) versus non-glomerular (*n* = 180) patches in treated samples. **d**, Histogram of distances between immune cells and nearest podocyte. **e**, MPH analysis. (i) Glomerular patches. Scale bars, 0.2 mm. (ii) TopACT spot-level immune annotations. Scale bars, 20 μm. (iii) Single-patch MPH landscapes. (iv) Average MPH landscapes over all patches. Treated average indicates large peripheral loop structures (compare Fig. 1f(i) and (v)). **f**–**h**, Multiplex immunofluorescence analysis (three control, three treated kidneys). **f**, Representative renal cortex immunofluorescence. Scale bars, 100 μm. **g**, Mean immune intensities in glomerular versus non-glomerular regions, *n* = 75 regions per region type and condition. Box plots, centre line shows mean; box limits show IQR; whiskers show full range. Increase (*P* = 5.6 × 10^−12^) in glomerular regions of treated samples consistent with TopACT. **h**, Ratio of mean intensity in outer to central glomerular region, by condition, for immune subtypes. Increased ratio (*P* = 7.3 × 10^−5^, *n* = 90 glomeruli per condition) over all immune types in treated samples consistent with MPH prediction. Individual increases for T cells (*P* = 2.7 × 10^−6^, *n* = 45 glomeruli per condition) and myeloids (*P* = 2.3 × 10^−3^, *n* = 75 glomeruli per condition). All statistical tests are one-sided Welch’s *t-*tests. NS (not significant), *P* ≥ 0.06, *P ≤ 0.05, **P ≤ 0.01, ***P ≤ 0.001, ****P ≤ 0.0001. Source Data

**Extended Data Fig. 2:**
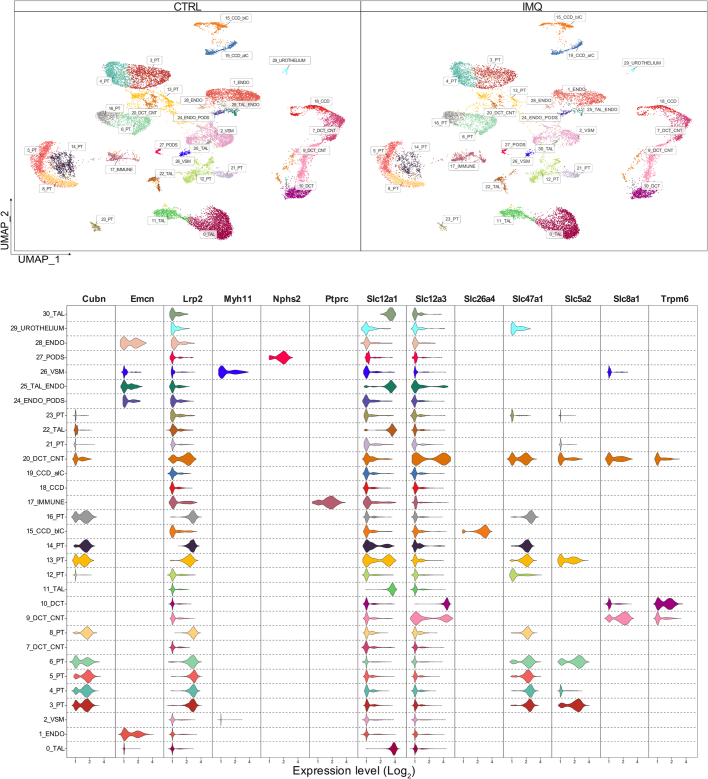
Reference snRNA-seq data for control and IMQ-treated mouse kidney. Top: UMAP embeddings with labelled cell type clusters. Bottom: Violin plots displaying highly differentially expressed genes per cell type. TAL = thick ascending limb; ENDO = endothelium; PODS = podocyte; VSM = vascular smooth muscle; PT = proximal tubule; DCT = distal convoluted tubule; CNT = connecting tubule; CCD = cortical collecting duct; CCD_aIC = cortical collecting duct α intercalated cell; CCD_bIC = cortical collecting duct β intercalated cell.

**Extended Data Fig. 3:**
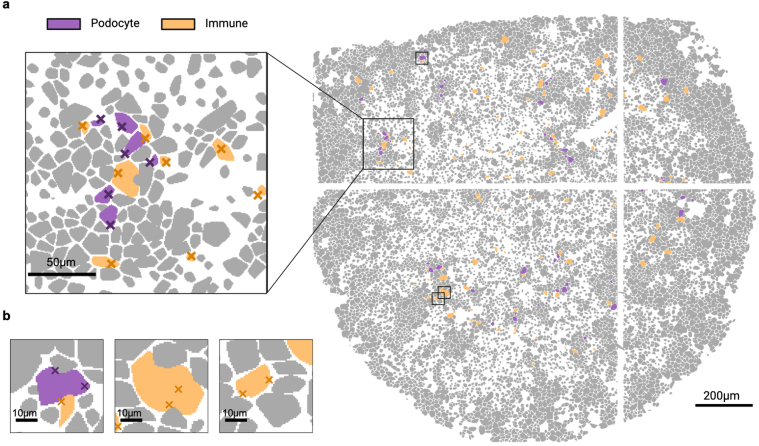
Concordance of TopACT predicted cells with ssDNA-based cell segmentation in a representative IMQ-treated mouse kidney sample. **a**, ssDNA-based cell segmentation annotated with TopACT-predicted immune (orange) and podocyte (purple) cells. Grey regions indicate ssDNA cell bins without a corresponding TopACT annotation. Right: The whole sample. Left: A magnified representative patch. Crosses denote TopACT-predicted cell centres. Note that each cell bin in the representative patch contains at most one TopACT prediction. **b**, Magnification of the three ssDNA-bins with more than one assigned TopACT cell. In each case the bin is assigned two TopACT cells of the same type.

**Extended Data Fig. 4:**
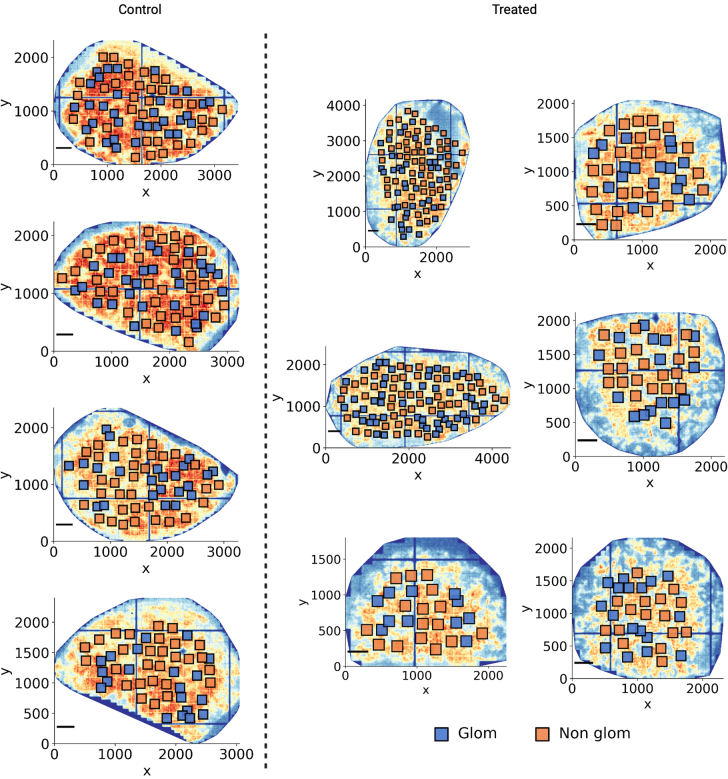
Mouse kidney patch distributions. Squares show glomerular (blue) and non-glomerular (orange) patches defined on each sample. Background heatmaps show smoothed transcript count. Scale bars: 0.2 mm.

Seeking to capture this behaviour spatially, we apply a version of TopACT trained to detect immune and podocyte cells to Stereo-seq data from a mouse kidney model (Fig. 5a and Extended Data Fig. 2). Mice treated with a toll-like receptor 7 (TLR7) agonist develop autoimmunity with kidney pathology modelling that observed in systemic lupus erythematosus, including mild renal immune infiltration (IMQ)^32^. The proposed method is able to resolve the precise locations of individual podocyte (Supplementary Methods 2 and Supplementary Fig. 3) and immune cells in space. In particular, comparison with single-stranded DNA (ssDNA)-based cell segmentation indicates that TopACT reliably localizes sparse cells (Methods and Extended Data Fig. 3). Comparing immune cell counts in glomerular versus non-glomerular regions of tissue (Fig. 5b and Extended Data Fig. 4) identifies a statistically significant increase (one-sided Welch’s *t*-test, *t* = 3.988, *P* = 4.2 × 10^−5^) in immune cell counts per patch in glomerular regions (*M* = 1.56, s.e.m. = 0.12, *n* = 161 patches) compared to non-glomerular regions (*M* = 0.95, s.e.m. = 0.09, *n* = 180 patches) in treated samples (Fig. 5c), consistent with lupus-like immune infiltration.

**Extended Data Fig. 5:**
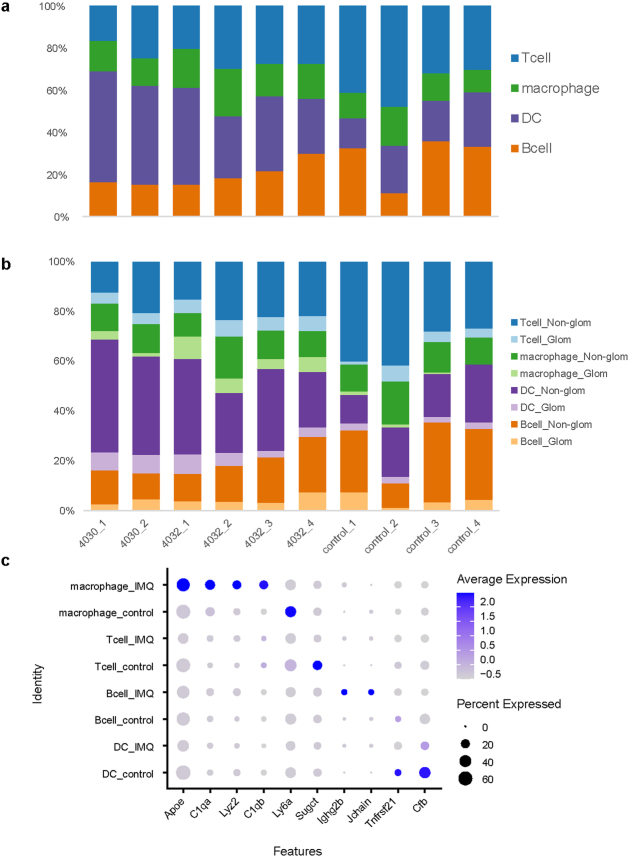
Analysis of spatial immune subpopulations in mouse kidney samples. **a**, Proportions of T-cells, macrophages, dendritic cells (DC) and B-cells across all samples. **b**, Proportions of T-cells, macrophages, dendritic cells (DC) and B-cells, split according to glomerular proximity. Glom = within glomerulus. Non-glom = not within glomerulus. **c**, Expression of immune subtype marker genes across immune subpopulations, separated by IMQ-treated and control. Macrophage markers: *Apoe*, *C1qa*, *Lyz2*, activated macrophage marker: *Ly6a*, T-cell marker: *Sugct*. B-cell markers: *Ighg2b*, *Jchain*. DC markers: *Tnfrsf21*, *Cfb*.

**Extended Data Fig. 6:**
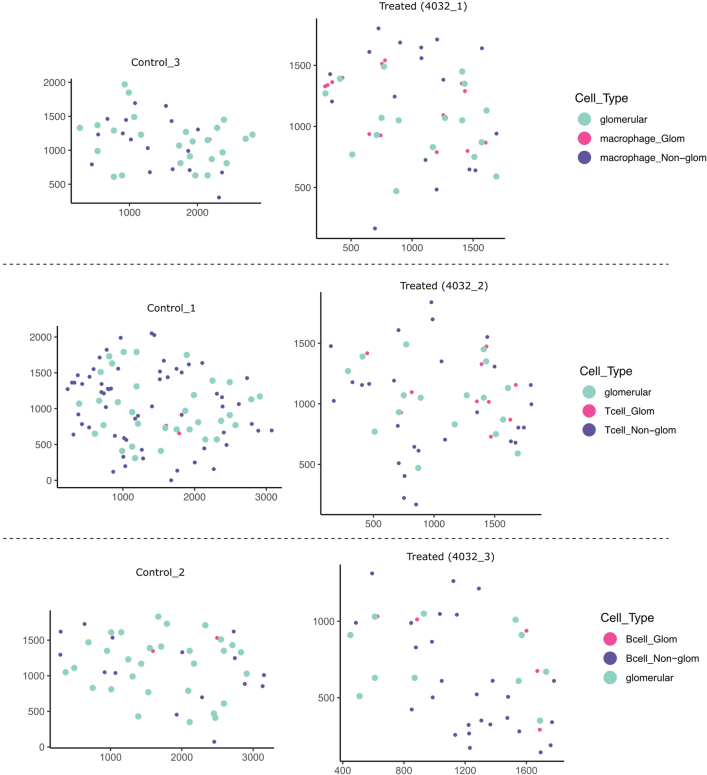
Spatial distribution of immune subpopulations relative to glomerulus loci. Top row: Macrophage in glomerulus (pink), macrophage outside of glomerulus (dark blue), glomerulus (pale blue). Middle row: T-cell in glomerulus (pink), macrophage outside of glomerulus (dark blue), glomerulus (light blue). Bottom row: B-cell in glomerulus (pink), B-cell outside of glomerulus (dark blue), glomerulus (light blue).

On the basis of immune subtype signatures from the snRNA-seq reference, we can infer the immune subtype for each TopACT-predicted immune cell with a supervised annotation. Subtype analysis shows that the renal immune expansion in treated mice (IMQ) is comprised of all subtypes except B cells and that a glomerular macrophage population is specific to the IMQ kidneys compared to controls (Methods and Extended Data Figs. 5 and 6). For further validation we also examine the ability of the method to identify tubule cells, in which we find that TopACT-predicted distal convoluted tubule and proximal tubule cells coincide with distributions of well-known marker genes for these cell types: Supplementary Methods and Supplementary Figs. 4–6.

For the purpose of renal pathology, a question, beyond the scope of multicellular-resolution spatial analysis, is whether the immune cells are in the circulation or have penetrated through to tissue. Analysis of TopACT classifications reveals that many infiltrating immune cells are located directly adjacent to neighbouring podocytes (101 immune cells across all IMQ-treated samples with centre-to-centre distance of, at most, 20 μm to a podocyte cell), providing strong evidence that these cells are penetrating, rather than separated by, the capillary endothelium (Fig. 5d). Consistent with this, tissue migration markers such as *Itga4*, *Itgal*, *Cd44* and *Spn* are found to be expressed in the snRNA-seq immune subclusters, indicative of infiltrating populations.

## MPH characterizes cell spatial patterns

Kidney architecture is highly complex and although existing spatial methods can place large-scale changes such as disease-driven fibrotic signatures in tissue context^33^ and infer colocalization of renal immune cells, fine-scale mapping of infiltrating immune cells is hampered by both resolution and low detection of immune cells in spatial data^34^. The ability of TopACT to spatially pinpoint immune cells now enables the systematic quantification of their spatial organization. MPH landscapes^9^ have proved highly successful in the study of the spatial organization of immune cells in tumours^7^. Inspired by this work, we compute MPH landscapes from the output of TopACT on small patches encapsulating each glomerulus in each sample. By taking the mean landscape across each sample, we arrive at an average summary of the multiscale immune cell topology around each glomerulus (Fig. 5e). We observe that the average MPH landscape corresponding to treated kidneys is activated at high radius parameters, indicating the presence of large loops of immune cells. This leads to the hypothesis that this behaviour is caused by the presence of a peripheral ring structure in immune cells infiltrating glomeruli in treated kidneys. We emphasize that this hypothesis is driven entirely by systematic analysis of spatial transcriptomics data.

To validate the predicted immune distributions at glomeruli, we performed multiplex immunofluorescence imaging with markers for T cell (CD4/CD8), B cell (CD19) and myeloid (CD11b, CD68 and GR1) lineages in Imiquimod-treated and control kidney sections from three control and three treated mice (Fig. 5f). Treated kidney showed an increase (one-sided Welch’s *t*-test, *t* = 7.438, *P* = 5.6 × 10^−12^) in the sum of the mean intensity across CD4/CD8 (T cells), CD19 (B cells) and CD11b (myeloid) in glomerular regions (*M* = 21.7, s.e.m. = 0.283, *n* = 75 regions) compared to non-glomerular regions (*M* = 18.0, s.e.m. = 0.401, *n* = 75 regions), whereas there was no difference in immune intensity between glomerular and non-glomerular regions in the control kidneys, consistent with the TopACT prediction (Fig. 5g). Moreover, quantification of peripheral glomerular versus central glomerular sum of mean intensity across CD4/CD8, CD19 and CD11b showed an increased ratio (one-sided Welch’s *t*-test, *t* = 3.917, *P* = 7.3 × 10^−5^) in treated glomeruli (*M* = 1.46, s.e.m. = 0.0571, *n* = 45 glomeruli) compared to control glomeruli (*M* = 1.21, s.e.m. = 0.0271, *n* = 45 glomeruli) (Fig. 5h), consistent with the MPH landscapes prediction of a peripheral ring structure. Considering the individual cell types, both T cells and myeloid cells, but not B cells, exhibited peripheral glomerular enrichment in treated kidneys (Fig. 5h), confirming the spatial immune subtype profiles predicted by TopACT (Extended Data Figs. 5 and 6).

## Discussion

In this work, we have introduced, implemented and applied TopACT, a multiscale method for topological automatic cell type classification. The proposed approach resolves cell type information at subcellular resolution and zeros in on the location of elusive sparsely dispersed cells. By replacing the fixed-window view with a flexible, multiscale lens, TopACT achieves significantly higher accuracy in subcellular spatial cell type identification than does the naive fixed-window approach. We showcased the strengths of this approach on mouse brain and kidney data generated by Stereo-seq^6^, which offers nanoscale-resolution, whole-transcriptome measurement of gene expression. Validating the method, predicted cells map to nuclear imaging and to the locations of cell type-specific markers. The recent launch of the Visium HD technology^4^ indicates a demand for subcellular spatial methods that can provide whole-transcriptome data, as important tools for discovery science. Complementary to these hypothesis agnostic tools, spatial methods using targeted panels of genes can achieve high capture efficiency, enabling confident validation of candidate genes and pathways. We show that TopACT is applicable to both and can address limitations in imaging-based cell segmentation in the Xenium technology, generating more faithful morphology and improving the accuracy of cell annotation. We leveraged the high-dimensional, fine-scale detail of Stereo-seq to locate individual immune cells by subtype and by measuring immune cell proximity to podocytes can infer glomerular tissue infiltration. By integrating TopACT with MPH landscapes^7–9^, we showed the spatial arrangement of glomerular immune cells in lupus nephritis, demonstrating the effectiveness of the topological approach in quantifying and elucidating tissue organization. TopACT is a highly general and flexible mathematical methodology, offering a powerful approach to the problem of expression aggregation in the absence of cell boundaries. We envisage that combining topological analysis based on RNA transcript signatures, with imaging-based nuclear and cell membrane information, would further improve the present TopACT method. In future, the proposed method can be applied directly to higher-dimensional data including spatiotemporal and three-dimensional data, as experimental technologies evolve in this direction.

## Methods

### TopACT

TopACT operates on subcellular-resolution spatial transcriptomics data. These data take the form of a grid of spots such that each spot in the grid has an associated expression vector in ${\mathbb{R}}$^*D*^. The output of our method is a cell type annotation for each spot in the grid. First, we use an annotated sc/snRNA-seq reference dataset to construct an automatic single-cell classifier, called a local classifier. This classifier could in principle use any supervised learning approach; for example, a neural network or random forest but a simple and effective choice is the support vector machine (SVM)^35–37^. The only restriction is that the classifier must take as input an expression vector and output a probability vector over all cell types. We achieve this by using Platt scaling^38^. Given a local classifier, TopACT then proceeds as follows. Fix a spot *s* in the grid. For a given radius *r* ≥ 0, consider a ball of radius *r* drawn about *s*. Let **X**_*r*_ be the sum of all the expression vectors of spots in this ball. Feeding **X**_*r*_ as input to the local classifier produces a probability vector **v**_*r*_ over the cell types. For a list *r*_1_ ≤ *r*_2_ ≤ … ≤ *r*_*k*_ of radii, these vectors can be combined into a multiscale cell type confidence matrix *A* = [${{\bf{v}}}_{{r}_{1}}$, ${{\bf{v}}}_{{r}_{2}}$, …, ${{\bf{v}}}_{{r}_{k}}$]. Now, pick a confidence threshold *θ* ∈ (0, 1). For a spot *s*, let *j* be minimal such that the most likely cell type *i* at scale *r*_*j*_ has confidence *A*_*ij*_ ≥ *θ*. We set the cell type of *s* to be *i* if such a *j* exists. In other words, the cell type assigned to the spot *s* is the cell type predicted by the local classifier at the smallest possible scale at which a confident prediction can be made. For a full technical description of the TopACT method, see Supplementary Methods 1A.

### Synthetic data generation

We use a two-stage process to generate synthetic benchmark data, first generating a synthetic cell type map and then imputing gene expression. A synthetic grid of spots with cell type annotations is produced. We sample 625 points uniformly at random from the unit square [0, 1] × [0, 1], taking these points to be cell centres. We draw a Voronoi diagram (computed using the implementation in SciPy^39^ based on Qhull^40^) on the basis of these points to simulate cell boundaries. Cell types are then assigned at random to each Voronoi region, in proportion to the cell type abundances in the snRNA-seq data. These cell types are then applied to a 500 × 500 grid of spots overlaid on the unit square. The end result is a grid of spots, each annotated with a cell type. Next, we impute the gene expression at each spot using a Poisson process with parameters inferred from the mouse kidney snRNA-seq data described below. This process is based on a simplified version of the model described by ref. ^24^. In detail, for a cell type *T* and gene *g* let *λ*_*Tg*_ denote the mean expression of gene *g* over all cells in the snRNA-seq dataset with cell type *T*. If a spot *s* is assigned the cell type *T*, we then model the expression **v**_*sg*_ of gene *g* at *s* as being distributed by a Poisson distribution

$${{\bf{v}}}_{sg} \sim {\rm{Poisson}}(\alpha {\lambda }_{Tg})$$

where *α* = exp(−7.3) is a fixed parameter determining the transcriptional abundance. To model zero-inflation, we then select 20% of spots uniformly at random to be assigned zero reads, regardless of the Poisson-modelled expression.

For molecular diffusion experiments we modelled the diffusion effect separately for each synthetic transcript. Given a transcript *a*, we sampled a diffusion magnitude *D*_*a*_ *~* exp(*λ*_diff_) from an exponential distribution where *λ*_diff_ is the mean diffusion for a single transcript. Independently, a diffusion direction *θ*_*a*_ is sampled from a uniform distribution over [0, 2π). This yields coordinate-wise displacements

$${d}_{a}^{x}={D}_{a}\cos {\theta }_{a}\,{d}_{a}^{y}={D}_{a}\sin {\theta }_{a}$$

The original spot coordinates *x*_*a*_, *y*_*a*_ for the transcript can then be revised accordingly to displaced coordinates

$${x}_{a}^{{\rm{d}}{\rm{i}}{\rm{f}}{\rm{f}}}={x}_{a}+\lfloor {d}_{a}^{x}/{d}_{{\rm{s}}{\rm{p}}{\rm{o}}{\rm{t}}}\rfloor \,{y}_{a}^{{\rm{d}}{\rm{i}}{\rm{f}}{\rm{f}}}={y}_{a}+\lfloor {d}_{a}^{y}/{d}_{{\rm{s}}{\rm{p}}{\rm{o}}{\rm{t}}}\rfloor $$

The rescaling by *d*_spot_ = 0.715 accounts for the inter-spot distance of 0.715 μm in Stereo-seq mouse kidney experiments.

### Synthetic data analysis

We ran TopACT directly on synthetic data, with an SVM local classifier trained on the same snRNA-seq reference dataset used for generation. For fixed-window bin 20 analysis, we split the 500 × 500 synthetic grid into square bins, each covering a 20 × 20 region of spots. Bin 20 was chosen so that each bin matches the mean area of a synthetic cell. Moreover, at bin 20 the resulting grid approximates 10 μm resolution, which is considered the ‘sweet spot’ for single-cell analysis^1^. We then summed the expression over all spots in each region. RCTD^24^ was run on couplet mode with default settings, using the same snRNA-seq reference dataset and we assigned each bin the RCTD predicted ‘first type’. For the modal cell type classification, we assigned to each bin its most frequent ground truth cell type. For analysis of rare cell type identification, we took rare cell types to be those making up less than 5% of the total samples in the snRNA-seq reference data. To extract cell loci, we first computed a binary image corresponding to each rare cell type. We performed a binary dilation to clean any cells that were split into several adjacent components and then took the centre of each connected component to be a cell centre.

### MPH landscapes

MPH tracks how the topological features (here, loops) of a shape evolve as certain parameters are varied. Given an input point cloud, we record the first persistent homology (*H*_1_) of its associated Rips-codensity bifiltration. This information is summarized in a sequence, called an MPH landscape^9^, of functions *λ*_*k*_: ${\mathbb{R}}$ ×  ${\mathbb{R}}$ → ${\mathbb{R}}$ for *k* = 1, 2,…. Given a radius parameter *s* and a codensity parameter *t*, the value *λ*_*k*_(*s*, *t*) ∈ ${\mathbb{R}}$ roughly describes the significance of the *k*th most significant topological feature in the bifiltration at those parameter values. Here, we focus on *λ*_1_: ${\mathbb{R}}$ × ${\mathbb{R}}$ → ${\mathbb{R}}$ which describes the significance of the most significant such feature. For a full introduction to MPH, see Supplementary Methods 1B. Here, we computed average control and treated MPH landscapes for TopACT-predicted immune cell points clouds (Supplementary Methods 2B). MPH was computed with RIVET (https://github.com/rivetTDA/rivet/) and converted to MPH landscapes using the code from ref. ^7^ (https://github.com/MultiparameterTDAHistology/SpatialPatterningOfImmuneCells).

### Statistical tests and reproducibility

Statistical tests were performed using the Python packages Scipy (https://www.scipy.org/) and statannotations (https://github.com/trevismd/statannotations). All tests are one-sided Welch’s *t*-tests.

The snRNA-seq (one control and two lupus nephritis mice, four healthy human kidney samples) and spatial experiments (one control and two lupus nephritis mice, one IgAN patient kidney sample) were not replicated. Mouse histological images are representative of images from six lupus and six control animals and multiplex immunofluorescence images representative of images from three control and three lupus mice.

### Animals

Female BALB/cOlaHsd mice were purchased from Envigo at 5 weeks of age. Animals were housed in specific pathogen-free individually ventilated cages, at 20–24 °C and 45–65% humidity, kept on a 12 h light/dark cycle from 08:00 to 20:00, with food and water freely available. All animal experiments were performed under project licence P84582234, with UK Home Office approval and local approval by the Oxford University Clinical Medicine Animal Welfare and Ethical Review Body and were carried out in compliance with UK Home Office Guidelines and the Animals Scientific Procedure Act 1986 (amended 2013) and reported in line with the ARRIVE guidelines. Mice were treated topically with either 5% Aldara (Imiquimod) cream (Meda Pharmaceuticals) or Vaseline (Unilever) control on both ears, three times weekly for 8 weeks.

### Mouse kidney processing

Snap-frozen mouse kidneys were obtained from 12 female mice randomized to treatment TLR7 agonist or control (unblinded). Treated mice develop lupus-like renal disease with glomerular endocapillary proliferation, which includes proliferation of circulating immune cells that have migrated to the capillary tuft. The 10 μm cryosections from three mice (four slices from a control sample and two and four slices, respectively, from two treated samples) were successfully processed for spatial transcriptomics with Stereo-seq^6^. Remaining kidney tissue from the above samples was dissociated to single nuclei, partitioned and sequenced to generate snRNA-seq data^41^, yielding a matched single-nucleus and subcellular spatial transcriptomics dataset. We cluster cells in the snRNA-seq dataset using Seurat^19^ and annotate cell types according to top marker genes. Initial clustering identified 30 populations, shown in Extended Data Fig. 2, alongside key cell type markers. The spatial data for each sample consist of gene expression readings measured on a grid of 220 nm DNA nanoball spots with a centre-to-centre inter-spot distance of 715 nm. These data are represented by a *D*-dimensional expression vector (*D* ≈ 25,000) at each spot. Spatial analysis was restricted to a boundary region defined as the convex hull of high expression spots (Supplementary Methods 2 and Supplementary Fig. 1).

### Human tissue

We used the Xenium platform (10x Genomics)^17^ to generate spatial data from a human kidney IgA nephropathy biopsy core obtained with the assistance of the Oxford Centre for Histopathology Research as an approved project under the Oxford Radcliffe Biobank research tissue bank ethical approval (South Central—Oxford C Research Ethics Committee: 19/C/0193). For snRNA-seq, healthy control kidney tissue was obtained from pre-implantation biopsies in four living donor kidneys through an approved project in the Oxford Transplant Biobank (South Central—Oxford C Research Ethics Committee: 19/SC/0529). All human samples were obtained with informed patient consent for research. Human tissue experiments were carried out under the University of Oxford Human Tissue Act license 12217.

### Mouse brain model

We examined publicly available adult mouse spatial data profiled with Stereo-seq^6^. To ensure an unbiased comparison, for scRNA-seq integration we used the same reference mouse brain atlas^42^ as in the original spatial study and trained the TopACT local classifier to identify the PVM1 subcluster. Similar to mouse kidney, we restricted analysis to a boundary region defined as the convex hull of high expression spots. From TopACT spot classifications, we performed a binary dilation and then called PVM cells as connected components of size at least 60 spots. To validate these called cells, we examined the expression level of PVM marker genes (as defined in the same atlas^42^) in TopACT-predicted PVM cells compared to uniformly sampled background cells.

### Spatial RNA-seq

Stereo-seq was performed as previously described^6^. Capture chips were loaded with DNA nanoballs (DNB) generated by rolling circle amplification of random 25 base pair (bp) oligonucleotides. Single end sequencing (MGI DNBSEQ-Tx) was performed to determine the DNB coordinate identity at each spatial location on the chip, followed by ligation of 22 bp polyT and 10 bp molecular identity oligos to the DNB. The 10 μm kidney tissue sections were cryosectioned from optimal cutting temperature (OCT) embedded frozen blocks and adhered to the chip surface, fixed in methanol, stained with nucleic acid dye (Thermo Fisher Scientific, Q10212) for imaging and incubated at 37 °C with 0.1% pepsin (Sigma, P7000) for 12 min to permeabilize. After permeabilization, we performed reverse transcription and cDNA amplification and the Agilent 2100 was used to check the range of cDNA fragments. The cDNA was interrupted by inhouse Tn5 transposase and amplified and the fragments double-selected. After screening, libraries were subjected to Agilent 2100 quality inspection. Finally, the double-selection libraries were constructed into libraries suitable for the MGI DNBSEQ-Tx sequencing platform through circularization steps and were sequenced to collect data (50 bp for read 1 and 100 bp for read 2).

For Xenium (10X Genomics) on formalin fixed paraffin embedded human renal biopsy blocks, tissue was sectioned and 5 μm slides prepared following the manufacturer-recommended protocol (CG000580) onto Xenium slides using the predesigned 377 gene 10X Human Multi-Tissue and Cancer Xenium Pre-Designed Gene Expression Panel (10x, 1000626). Padlock probes were incubated on the tissue overnight before rolling circle amplification and chemical autofluorescence quenching. Slides were imaged on a Xenium Analyzer machine. Cell segmentation, gene transcript by cell and transcript by tissue location data matrices were generated by the Xenium Onboard Analysis pipeline. Supervised cell clustering was performed in Seurat^19^ by finding a set of anchors between the healthy human snRNA-seq dataset and the per cell Xenium data and using these to transfer labels to the Xenium segmentation defined cells.

For TopACT, transcripts with *Q*-score of 20 or more were selected and binned to 1 μm resolution before processing with the standard TopACT pipeline with a minimum radius of 2 μm, a maximum radius of 5 μm and a confidence level of 0.9. The local classifier was trained from the paired snRNA-seq dataset using the procedure detailed in Supplementary Methods 2A.

### snRNA-seq

Tissue from the same frozen organs used for mouse kidney spatial transcriptomics was used to perform complementary snRNA-seq. Single nuclei were isolated as previously described with minor modifications^43^. In brief, kidney tissues were placed into a 2 ml Dounce homogenizer (Sigma) with 2 ml of prechilled homogenization buffer (10 mM Tris pH 8.0 (Thermo Fisher), 250 mM sucrose (Sigma), 1% BSA (Sangon Biotech), 5 mM MgCl_2_ (Thermo Fisher), 25 mM KCl (Thermo Fisher), 0.1 mM dithiothreitol (Thermo Fisher), 1X protease inhibitor cocktail (Roche), 0.4 U μl^−1^ of RNase inhibitor (MGI), 0.1% NP40 (Roche)). After incubation on ice for 10 min, tissues were homogenized by ten strokes of the loose pestle A and filtered with 70 μm cell strainer (Falcon). The homogenate was further homogenized with ten strokes by tight pestle B, filtered using 30 μm cell strainer (Sysmex) into 15 ml conical tube and centrifuged at 500*g* for 5 min at 4 °C. The pellet was resuspended in 1 ml of blocking buffer (PBS (Thermo Fisher), 1% BSA, 0.2 U μl^−1^ of RNase inhibitor) and centrifuged at 500*g* for 5 min; this step was repeated once. The pellet was resuspended using cell resuspension buffer (MGI) at concentration of 1,000 nuclei per μl for further library preparation. The snRNA-seq libraries were prepared using DNBelab C Series Single-Cell Library Prep Set (MGI, no. 1000021082)^44^ Droplets were generated from a single nuclei suspension, followed by emulsion breakage, bead collection, reverse transcription and cDNA amplification to generate barcoded libraries. Indexed libraries were constructed following the manufacturer’s protocol, quantified using Qubit ssDNA Assay Kit (Thermo Fisher Scientific, Q10212) and sequenced using DNBSEQ-T1 at the China National GeneBank (Shenzhen, China) with read length 41 bp for read 1, 100 bp for read 2 and 10 bp for sample index.

For human snRNA-seq, single nuclei were isolated from fresh or liquid nitrogen flash-frozen renal biopsies using the 10X Genomics Chromium Nuclei Isolation Kit with RNase Inhibitor 16 rxns (PN-1000494), following the kit protocol with the following modifications, 0.2 U μl^−1^ of supplemental RNase inhibitor was added to the lysis buffer and debris removal buffer, polypropylene 1.5 ml Eppendorf collection tubes were coated with 10% BSA (MACS BSA Stock Solution, Miltenyi Biotec, 130-091-376) overnight before use and the final nuclei suspension was filtered through a 40 μm FLOWMI cell strainer (SP Bel-Art, 136800040). Samples with more than 1 million nuclei were then flow cytometry sorted to clean up the sample using Sytox-7AAD Live dead staining (Invitrogen S10349). Samples with less than 1 million nuclei after washing were filtered a second time with the 40 μm FLOWMI cell strainer. Gene libraries from isolated human renal single nuclei were constructed with droplet-based scRNA-seq using the Chromium Next GEM Single Cell 3′ GEM, Library & Gel Bead Kit v.3.1, 4 rxns (PN-1000128). Single nuclei were loaded at 1,000 nuclei per μl for a targeted yield of 10,000 nuclei per sample. Libraries were sequenced on a NovoSeq6000 (Illumina) at the Oxford Genomics Centre or with Novogene. Runs were demultiplexed and the resulting fastq files processed through the 10X Genomics Cellranger pipeline. Filtered gene matrix data were then analysed in R using the Seurat package.

### Single-nucleus clustering

The snRNA-seq data were analysed using Seurat v.4.0.2 (mouse) or 4.4.0 (human)^19^. Mouse nuclei were filtered on gene count less than 500 or greater than 3,500 and mitochondrial percentage greater than 5. Human nuclei were filtered and selected for gene features greater than 500 and less than 6,000, gene count less than 25,000 and mitochondrial percentage less than 25. Data were log normalized, variable features identified and linear transformation scaling performed. Principal component analysis dimensionality reduction was run before the human snRNA-seq data were Harmony^45^ integrated to remove batch effects. The first 30 principal components were selected and clusters identified using the ‘FindClusters’ method in Seurat with a resolution of 0.6 (mouse) or 0.5 (human). The ‘FindAllMarkers’ function was used to identify genes that characterized each cluster and differential expression of genes was tested between clusters. Cluster annotation was performed manually on the basis of the top markers, applying knowledge of renal physiology with reference to the literature. Visualizations of the annotated snRNA-seq dataset in the form of UMAP and violin plots are available in Extended Data Figs. 1 (human) and 2 (mouse).

### Mouse kidney immune subclustering

To investigate the subpopulations of TopACT-identified immune cells in mouse kidney, we performed a supervised annotation of these cells based on snRNA-seq subclusters using Sonar^46^. Annotation resolved subpopulations of B cells, dendritic cells, macrophages and T cells. Examining the proportions of these subpopulations across samples (Extended Data Fig. 5a) shows expansion of T cell, dendritic cell and macrophage populations in IMQ-treated kidneys. Annotating each immune cell according to its proximity to glomeruli further shows acquisition of T cells, dendritic cells and macrophage cells within the glomeruli themselves (Extended Data Fig. 5b). Extended Data Fig. 5c shows the expression of cell type-specific markers by immune subset and condition (IMQ-treated or control). We visualize the spatial distribution of these subpopulations for representative samples in Extended Data Fig. 6. This visualization shows enrichment of T cells (top row) and macrophages (middle row) but not B cells (bottom row), in glomeruli, consistent with multiplex immunofluorescence results in Fig. 5.

### Comparison to ssDNA-based segmentation

We used ssDNA imaging of the mouse kidney Stereo-seq samples to compute cell segmentation based on cell nucleus locations, as previously described^6^. For further validation, we compared TopACT-called cell loci for immune and podocyte cells with these cell bins on a single representative IMQ-treated sample. A TopACT-predicted cell was assigned to an ssDNA bin if its centre was in the bin itself or if the centre-to-centre distance of the bin was sufficiently close (within ten spots, 7.15 μm), the latter case dealing with the scenario of two cell bins with overlapping boundaries but non-coincident cell centres. We found that 110 out of 137 (80%) of TopACT-predicted immune cells and 46 out of 50 (92%) of TopACT-predicted podocyte cells coincided with an ssDNA bin. Extended Data Fig. 3 shows the cell bins annotated according to the assigned TopACT cell type. Only three ssDNA bins were found to coincide with more than one TopACT-predicted cell, providing further evidence that TopACT predictions correspond to ground truth cells. Visual inspection of these three examples is suggestive of the underlying ssDNA bins being doublets.

### Multiplex immunofluorescence

Multiplex immunofluorescence staining was performed on 4-μm-thick formalin fixed paraffin embedded sections according to the OPAL protocol (Akoya Biosciences) on the Leica BOND RXm auto stainer (Leica Microsystems). Six staining cycles were carried out using the following primary antibody–Opal fluorophore pairs; thereafter the sections were counterstained with diamidino-2-phenylindole (DAPI; FP1490, Akoya Biosciences): Ly6G/Ly6C (Gr-1) 1:400 (MAB1037-SP; R&D Systems)–Opal 480 1:150; CD4 1:500 (ab183685; Abcam)–Opal 520 1:150; CD8 1:800 (98941; Cell Signaling)–Opal 520 1:150; CD68 1:1,200 (ab125212; Abcam)–Opal 570 1:150; CD19 1:600 (90176; Cell Signaling)–Opal 620 1:150; CD11b 1:80,000 (ab133357; Abcam)–Opal 690 1:150; and E-cadherin 1:500 (3195; Cell Signaling)–Opal 780 1:25.

Samples were deparaffinized and rehydrated according to Leica BOND Rx protocol. Antigen retrieval was carried out at 100 °C for 20 min, tissue sections were incubated in either Epitope Retrieval solution 1 or 2, before the application of each primary antibody. Sections were incubated with primary antibody for 1 h; thereafter, Opal fluorophores were substituted for 3,3′‐diaminobenzidine (DAB) and added using the BOND Polymer Refine Detection System, with a 10 min incubation time. After completion of all cycles of staining, sections were counterstained with DAPI and mounted with VECTASHIELD Vibrance Antifade Mounting Medium (H-1700-10; Vector Laboratories). Slides were scanned and multispectral images of tissue sections obtained using the Akoya Bioscience Vectra PolarisTM. Analysis of the images was carried out using Zeiss Arivis v.4.1.1 (Zeiss). Mean intensity was measured across glomerular regions, manually defined on the basis of E-cadherin and nuclei and non-glomerular regions comprising the whole field of view with the glomerular regions subtracted. Mean intensities for each channel were summed per glomerular and non-glomerular region to calculate the total ‘immune’ intensity per glomerular region. Ratios were calculated per glomerulus using the mean intensity which was calculated by subtracting the mean intensity of a central glomerular region from the mean intensity of the whole glomerular region.

### Reporting summary

Further information on research design is available in the Nature Portfolio Reporting Summary linked to this article.

## Online content

Any methods, additional references, Nature Portfolio reporting summaries, source data, extended data, supplementary information, acknowledgements, peer review information; details of author contributions and competing interests; and statements of data and code availability are available at 10.1038/s41586-024-07563-1.

### Supplementary information

Supplementary Information Supplementary Methods 1 and 2, Figs. 1–6 and references.

Reporting Summary

### Source data

Source Data Fig. 2

Source Data Fig. 3

Source Data Fig. 5

## Data Availability

The immunofluorescence images are available through the Oxford University Research Archive at 10.5287/ora-b7ob4g8ba. Raw Fastq files for Stereo-seq and snRNA-seq in mouse lupus and control are available in the NCBI Sequencing Read Archive (SRA) with BioProject accession PRJNA1099448. The following datasets are deposited in the NCBI Gene Expression Omnibus (GEO): the Seurat object from the mouse snRNA-seq with accession GSE265819, the bin 1 matrix generated from the Stereo-seq dataset with accession GSE264321, raw Fastq and Seurat object for human kidney healthy snRNA-seq with accession GSE264393, Xenium data from human IgAN kidney with accession GSE264334. Mouse brain spatial data were obtained from https://db.cngb.org/stomics/mosta/ and corresponding brain scRNA-seq from the NCBI SRA with accession SRP135960. Output files for TopACT and related analysis are available at Zenodo (10.5281/zenodo.10950538)^47^. Source data are provided with this paper.

## Code availability

The Python implementation of TopACT used in this work is available at GitLab (https://gitlab.com/kfbenjamin/topact) and archived at Zenodo (10.5281/zenodo.11050773)^48^. Code for the experiments in this paper are available at Zenodo (10.5281/zenodo.11050996)^49^.
