# Supplementary material for: Multiscale topology classifies cells in subcellular spatial transcriptomics
Source: Nature. 2024 Jun 19;630(8018):943–9. doi: 10.1038/s41586-024-07563-1 (PMC11208150; doi:10.1038/s41586-024-07563-1)
Supplement: Supplementary file 1 — Supplementary Methods 1 and 2, Figs. 1–6 and references. [file 41586_2024_7563_MOESM1_ESM.pdf]

nature portfolio

https://doi.org/10.1038/s41586-024-07563-1

Supplementary information

Multiscale topology classifies cells in subcellular spatial transcriptomics

In the format provided by the authors and unedited

Supplementary Information for *Multiscale topology classifies cells in subcellular spatial transcriptomics*

Katherine Benjamin[1*], Aneesha Bhandari[2,3*], Jessica D. Kepple[2,3], Rui Qi[2,3], Zhouchun Shang[4,5], Yanan Xing[4,5], Yanru An[4], Nannan Zhang[6], Yong Hou[4], Tanya L. Crockford[2,3], Oliver McCallion[7], Fadi Issa[7], Joanna Hester[7], Ulrike Tillmann[1,8], Heather A. Harrington[1,2,9,10,11✉] & Katherine R. Bull[2,3✉]

[1]Mathematical Institute, University of Oxford, Oxford OX2 6GG, United Kingdom. [2]Centre for Human Genetics, University of Oxford, Oxford OX3 7BN, United Kingdom. [3]Nuffield Department of Medicine, University of Oxford, Oxford OX3 7FZ, United Kingdom. [4]BGI Research, Riga 49276 Latvia. [5]College of Life Sciences, University of Chinese Academy of Sciences, Beijing, 100049, China. [6]BGI Research, Qingdao 266555, China. [7]Translational Research Immunology Group, Nuffield Department of Surgical Sciences, University of Oxford, Oxford OX3 9DU, United Kingdom [8]Isaac Newton Institute for Mathematical Sciences, University of Cambridge, Cambridge CB3 0EH, United Kingdom. [9]Max Planck Institute of Molecular Cell Biology and Genetics, 01307 Dresden, Germany. [10]Centre for Systems Biology, Dresden, 01307 Dresden, Germany. [11]Faculty of Mathematics, Technische Universität Dresden, 01062 Dresden, Germany.

*Equal contribution ✉e-mail: harrington@maths.ox.ac.uk, katherine.bull@ndm.ox.ac.uk

Supplementary Methods 1: Analysis techniques 1

A Cell type classification for subcellular spatial transcriptomics 1
- A.1 Subcellular spatial transcriptomics model 1
- A.2 TopACT method description 1

B Multiparameter persistent homology 4
- B.1 Single parameter persistent homology 4
- B.2 Multiparameter persistence 4
- B.3 MPH landscapes 5

Supplementary Methods 2: Data analysis 6

A TopACT implementation 6
- A.1 Gene filtering 6
- A.2 Learning a local classifier 6
- A.3 Hyperparameters 6

B Stereo-seq data analysis 6
- B.1 Defining sample boundaries 6
- B.2 TopACT output 6
- B.3 Detecting single cells from TopACT output 6
- B.4 Validation via podocyte prediction 7
- B.5 Validation on tubule cells 7
- B.6 Generating patches 7
- B.7 MPH landscapes 7

Supplementary References 7

Supplementary Figures 9

Supplementary Fig. 1 | Definition of sample boundaries based on transcript density

Each axis shows a single sample (Left: Control. Right: Treated). Black dashed lines show convex hulls of high-density regions in each sample, which are used to restrict later analysis. Background heatmaps show smoothed transcript count. Scale bars = 0.2mm.

Supplementary Fig. 2 | Image analysis pipeline for detecting cells and glomeruli

(A) Extracting cell loci from spot-level cell type predictions. First, a binary image is produced indicating spots assigned the given cell type. Then, Gaussian smoothing is applied to produce a grayscale image. Finally, DoG blob detection is used to detect regions of high density of the given cell type. These regions are taken as predicted cell loci. In this example, immune cell loci are detected. **(B)** Extracting ground truth glomerulus loci from Bin 20 cell type predictions. The pipeline is identical to that set out in (A), but run specifically on podocyte predictions at Bin 20.

Supplementary Fig. 3 | Comparison of TopACT podocyte predictions to ground truth

TopACT predicted podocyte cells (blue cross) and ground truth glomeruli (black square) for each sample. Note that predicted podocytes colocalise with glomeruli, as expected, validating the use of TopACT on mouse kidney data. Dashed black lines show samples boundaries as in Supplementary Fig. 1. Scale bars = 0.2mm

Supplementary Fig. 4 | Marker gene expression in predicted mouse kidney cells

Violin plots show expression of common markers of proximal tubule (PT) distal convoluted tubule (DCT), and podocyte cells, for TopACT predicted PT cells (blue), TopACT predicted DCT cells (green), TopACT predicted podocyte cells (purple), and randomly sampled background cells (grey), across all mouse kidney samples. Each plot corresponds to the expression counts of a single given marker gene in cells labelled with the given cell type across all samples. Top rows: PT markers. Bottom row, first half: DCT markers. Bottom row, second half: podocyte markers. Log scale.

Supplementary Fig. 5 | Comparison of TopACT distal convoluted tubule (DCT) predictions to spatial distribution of marker genes

TopACT-predicted DCT cells (black cross) overlaid on map of combined density of DCT marker genes (blue background). Markers are *Slc12a3*, *Trpm6*, *Egf*, and *Aqp2*. Note that predicted DCT cells are found in areas of high marker gene expression as expected. Dashed black lines show samples boundaries as in Supplementary Fig. 1. Scale bars = 0.2mm.

Supplementary Fig. 6 | Comparison of TopACT proximal tubule (PT) predictions to spatial distribution of marker genes

TopACT predicted PT cells (black cross) overlaid on map of combined density of PT marker genes (blue background). Markers are *Slc34a1*, *Lrp2*, *Cubn*, *Slc47a1*, *Slc5a12*, *Slc22a8*, *Slc5a2*, *Slc13a1*, and *Slc23a1*. Note that predicted PT cells are found in areas of high marker gene expression as expected. Dashed black lines show samples boundaries as in Supplementary Fig. 1. Scale bars = 0.2mm.

Supplementary Methods 1: Analysis techniques

Here we describe in detail the topological methods used in the main text. We start by introducing TopACT, a method for topological automatic cell type identification on subcellular spatial transcriptomics data. We then describe multiparameter persistent homology (MPH) landscapes, a method from Topological Data Analysis which we use to detect topological features in TopACT predicted immune cell distributions.

A. Cell type classification for subcellular spatial transcriptomics.

We begin by describing mathematical model for subcellular spatial transcriptomics data. We then detail how TopACT can be applied on such data to extract cell type classifications at the spot level.

A.1. Subcellular spatial transcriptomics model.

We begin by abstracting the notion of a spatial transcriptomics experiment. A general (non-spatial) transcriptomics experiment can be thought of as a collection of objects (for example, in single-cell transcriptomics the objects are cells), each equipped with a cell type t and an expression vector $v \in \mathbb{R}^D$. The vector v measures the number of reads in each of the D genes in the genome $\mathcal{G}$, and it is assumed that these are sampled from random variables corresponding to the cell type t. The key difference in a spatial transcriptomics experiment is that the objects are now equipped with a notion of distance, giving rise to a metric space. The present section formalizes this notion.

Experimental setup We begin with the following preliminary objects:

1. A metric space X called a *sample*;
2. A finite subset $\mathcal{S} \subset X$ of *spots*;
3. A finite ordered set $\mathcal{G} = \{g_1, \ldots, g_D\}$ of D *genes*;
4. A finite ordered set $\mathcal{T} = \{t_1, \ldots, t_K\}$ of K *cell types*.

These items together can be seen as a mathematical abstraction of a typical spatial transcriptomics experimental setup: we aim to measure the expression of each gene in $\mathcal{G}$ across the sample X, by taking readings from each spot in $\mathcal{S}$. These readings are determined by the underlying cell type in $\mathcal{T}$ associated to each spot.

In this setting, an experimental reading can be thought of as an assignment of an expression $v_{sg} \in \mathbb{R}$ for each spot $s \in \mathcal{S}$ and gene $g \in \mathcal{G}$. Equivalently, making use of the ordering on $\mathcal{G}$, we have a map

$$v \colon \mathcal{S} \to \mathbb{R}^D, \tag{1}$$

where $v(s)_i = v_{sg_i}$ for each $s \in \mathcal{S}$ and $1 \le i \le D$.

Expression model We now describe our model for how these expression assignments arise in practice. Underlying each experiment, we assume there is a set $\mathcal{T}$ of disjoint *cell types*, and that for each cell type $t \in \mathcal{T}$ and gene $g \in \mathcal{G}$ there is a corresponding random variable V_{tg} giving the count of the gene g measured in a cell of type t.

A subcellular spatial transcriptomics experiment can be seen as a partial assignment

$$\tau \colon X \rightharpoonup \mathcal{T} \tag{2}$$

of a cell type to some of the points in X. Given such an assignment, for each point $x \in \operatorname{dom} \tau$ we model

$$v_{xg} \sim V_{\tau(x)g}, \tag{3}$$

and we can assign $v_{xg} = 0$ whenever $x \notin \operatorname{dom} \tau$. Restricting these values to spots in $\mathcal{S}$, we recover an experimental reading.

We emphasize that in this subcellular model, each spot is assigned *at most* one cell type. In the case of multicellular spatial transcriptomics, as seen with data produced by e.g. ST/Visium (1) and Slide-Seq(v2) (2, 3), this assumption will not hold, as each spot records transcripts from multiple distinct cells.

A.2. TopACT method description.

Given that the expression vector $v(x) \in \mathbb{R}^D$ assigned to a point $x \in X$ depends on its cell type $\tau(x)$, a natural objective is to deduce the cell type map τ given the expression map v. In the case of single cell transcriptomics, where each expression vector contains sufficient information to deduce a cell type, this is a relatively straightforward task. In contrast, subcellular spatial data typically suffer from very low read counts, and it is therefore necessary to aggregate readings from neighboring spots in order to recover enough information to reliably predict a cell type. However, it is not clear how best to perform this aggregation without prior knowledge of cell boundaries.

Our approach is to assume that there exists a local neighborhood around each spot that belongs entirely to a single cell type. By combining the expression readings from this neighborhood, one obtains a pseudo-single-cell reading that can be classified by existing techniques. This yields a classification for each individual spot. The challenge now is to identify the correct scale at which to draw the neighborhood, and we resolve this by taking a 'multiscale' approach.

Local classifier definition Let T be a $\mathcal{T}$-valued random variable and Q a positive-integer-valued random variable. We are going to study a random variable describing the aggregated gene expression of Q spots that all assigned the cell type T.

For any cell type $t \in \mathcal{T}$ let

$$V_t = (V_{t,g_1}, \ldots, V_{t,g_D}) \tag{4}$$

be the $\mathbb{R}^D$-valued random variable describing the total expression over all genes of the cell type t. Then, if $V_t^1, \ldots, V_t^K$ are i.i.d copies of V_t, set $\Sigma V_t = \sum_{i=1}^{Q} V_t^i$ and

$$Z = \Sigma V_t / \|\Sigma V_t\|_1. \tag{5}$$

Z is therefore the normalized sum of Q expression readings independently drawn from the cell type t.

We say that a *local classifier* is any method that estimates the probability of each cell type given an observed normalized expression reading. More specifically, recalling that the cell types have an ordering $\mathcal{T} = \{t_1, \ldots, t_K\}$, we say that a local classifier is a function

$$f \colon \mathcal{Z} \to [0,1]^K, \tag{6}$$

where $\mathcal{Z} = \{z \in [0,1]^D : \|z\|_1 = 1\}$, such that

$$f(z)_i \approx \mathbb{P}(T = t_i \mid Z = z) \tag{7}$$

for all $1 \leq i \leq K$.

Producing a local classifier from sc/snRNA-seq data We can use single-cell or single-nucleus reference data sets to estimate the effect of the different cell types on the gene expression behavior and produce a local classifier. In detail, we take a collection $\mathcal{C}$ of single cell samples along with a gene expression map

$$v^{\mathrm{sc}} \colon \mathcal{C} \to \mathbb{R}^D$$

and a cell type map

$$\tau^{\mathrm{sc}} \colon \mathcal{C} \to \mathcal{T}.$$

From this information, we seek a classifier that takes as input normalized expression vectors and outputs probability distributions over the cell types in $\mathcal{T}$. To do this, we normalize each expression vector:

$$z^{\mathrm{sc}}(c) = \frac{v^{\mathrm{sc}}(c)}{\|v^{\mathrm{sc}}(c)\|_1}. \tag{8}$$

The input-output pairs $(z^{\mathrm{sc}}(c), \tau^{\mathrm{sc}}(c))$ then form training data for any standard supervised learning platform. In our case, we use a linear support vector machine (SVM) (4, 5) and estimate probabilities with Platt scaling (6) to produce a local classifier f.

Multiscale confidence matrix Let f be a local classifier. In order to classify a point $x \in X$ it may be necessary to aggregate expression readings around x. Write $B(x,r) = \{y \in X : d(x,y) \leq r\}$ for the closed ball of radius r in X centered on x. We define the aggregated gene expression

$$v(x,r) = \sum_{s \in B(x,r) \cap \mathcal{S}} v(s) \in \mathbb{R}^D, \tag{9}$$

and, if this is non-zero, set

$$z(x,r) = v(x,r)/\|v(x,r)\|_1 \in \mathcal{Z}. \tag{10}$$

In words, $z(x,r)$ describes the normalized gene expression about x at radius r. Then, if f is a local classifier as defined in Eq. (7), one obtains a probability vector

$$f(z(x,r)) \tag{11}$$

which can be interpreted as a cell type classification at the scale r.

Given an ordered collection $R = (r_1 \leq \cdots \leq r_L)$ of radii one then obtains a sequence of corresponding probability vectors, which can be combined into an $L \times K$ matrix $\mathfrak{M}^x$ defined by

$$\mathfrak{M}^x_{ij} = f(z(x, r_i))_j \tag{12}$$

which we call a *multiscale confidence matrix*. Here $\mathfrak{M}^x_{ij}$ records the confidence in cell type t_j at scale r_i around the point x.

Extracting cell type annotations Given a multiscale confidence matrix $\mathfrak{M}^x$, we would like to extract a cell type annotation for the spot x. The general principle followed by TopACT is that one should use the smallest scale possible to classify a point, because this minimizes the chance that the aggregated expression has been taken from surrounding cells of a different type.

Let $\theta \in [0,1]$ be a *confidence hyperparameter*. The *classification index* $i_\theta = i_\theta(x)$ of x is

$$i_\theta = \inf\{i \in \{1, \ldots, L\} : \|\mathfrak{M}^x_i\|_1 \geq \theta\}. \tag{13}$$

In other words, $r_{i_\theta(x)}$ is the lowest scale at which a cell type was predicted with confidence at least θ at the point x.

We now define the *TopACT predicted cell types* with respect to the collection R and confidence threshold θ:

$$\mathfrak{T}_{R,\theta} \colon X \rightharpoonup \mathcal{T}. \tag{14}$$

If $i_\theta(x) < \infty$ then we set $\mathfrak{T}_{R,\theta}(x)$ to be the cell type with the highest predicted probability at scale $r_{i_\theta(x)}$. Precisely, it is $\mathfrak{T}_{R,\theta}(x) = t_j$ where j maximizes the value of $\mathfrak{M}^x_{i_\theta(x)j}$.[1] If $i_\theta(x) = \infty$, i.e. if no scale produced sufficient confidence, then we do not specify a cell type. In other words, we have that $\operatorname{dom}\left(\mathfrak{T}_{R,\theta}\right) = \{x \in X : i_\theta(x) < \infty\}$.

Restricting TopACT to a square grid In the experiments considered in the manuscript, we work with either simulated or real-world Stereo-seq (7) data. For Stereo-seq experiments, we assume that spots are evenly spaced on a 2D square lattice. More precisely, we assume that the metric space X is a subspace of $\mathbb{R}^2$ and the set of spots is

$$\mathcal{S} = ([I] \times [J]) \cap X \tag{15}$$

for some $I, J \in \mathbb{N}$, where $[k] = \{1, \ldots, k\}$ for any $k \in \mathbb{N}$.

By further equipping $\mathbb{R}^2$, and therefore X, with the ℓ_∞ norm, it follows that the neighborhoods $B(x, r)$ are squares in X. In particular, for a spot $s \in \mathcal{S}$ the critical values $r_0 \leq r_1 \leq \ldots$ for which $B(x, r_i) \cap \mathcal{S}$ changes are precisely $r_i = i \in \mathbb{N}$. In this setting, then, we set $R = (0, 1, 2, \ldots, r_{\max})$ for some maximal radius parameter $r_{\max} \in \mathbb{N}$. Algorithm 1 demonstrates how to produce TopACT cell type annotations from these assumptions.

Algorithm 1 TopACT (Square grid)

Input: M, N: the dimensions of the spot grid;
V: an $M \times N \times D$ array where V_{ijk} is the expression of gene g_k at the spot (i, j);
$f \colon \mathcal{Z} \to [0,1]^K$: a local classifier;
θ: a confidence hyperparameter;
$r_{\max}$: the maximum radius.
Output: The TopACT cell type assignment $\mathfrak{T}_{(0,\ldots,r_{\max}),\theta} \colon [M] \times [N] \rightharpoonup \mathcal{T}$.

$\tau \leftarrow \emptyset$ ▷ An empty cell type assignment
for $s = (i, j) \in [M] \times [N]$ **do**
  $r \leftarrow 0 \in \mathbb{N}$
  $v \leftarrow 0 \in \mathbb{R}^D$
  while $r \leq r_{\max}$ and $s \notin \operatorname{dom} \tau$ **do**
    for all $s' = (i', j') \in [M] \times [N]$ such that $\|s - s'\|_\infty = r$ **do** ▷ Update pooled expression
      $v \leftarrow v + V_{i'j'}$
    if $v \neq 0$ **then**
      $z \leftarrow v / \|v\|_1$ ▷ Normalize expression for input to local classifier
      $k^* \leftarrow \operatorname{argmax}_{1 \leq k \leq K} f(z)_k$
      if $f(t)_{k^*} \geq \theta$ **then** $\tau(s) \leftarrow t_{k^*}$ ▷ Sufficient confidence to classify spot
    $r \leftarrow r + 1$ ▷ Increment radius to the next critical value
return τ

We remark that this setup may differ for different spatial transcriptomics technologies. For example, the spots may lie on a hexagonal grid as in HDST (8) or be randomly distributed as in Seq-Scope (9). Our method is general and applies equally to

[1] A tie between multiple cell types can be resolved by equipping the cell types with an order of precedence. Note that if $\theta > 0.5$ then a tie can never occur.

any such specification, including 3D or spatio-temporal data. TopACT only requires some notion of distance between the spots in $\mathcal{S}$.

B. Multiparameter persistent homology. Here, we provide a brief introduction to the theory of multiparameter persistent homology, a mathematical tool which tracks the presence of topological features in data as given parameter values are varied. In the case of a single parameter (where the parameter being varied is typically a type of scale), it is possible to represent this persistent homology via an interpretable summary known as a barcode. However, it is often the case that more than one parameter may need to be varied (here, to control for the presence of misclassifications), and in this case there is no complete, canonical description of the various topological information contained in the corresponding persistent homology. Nevertheless, there do exist interpretable summaries in this setting, and in this work we make use of the *multiparameter persistence landscapes* of Vipond (10). For a more complete theoretical discussion of multiparameter persistence see (11), and for a thorough exploration of applications to immune cell spatial distributions see (12).

B.1. Single parameter persistent homology. We give a brief overview of single-parameter persistent homology in order to motivate our use of the multiparameter generalization. See e.g. (13) for a more complete introduction. The aim of persistent homology is to extract information about the multiscale topology of a point cloud in $\mathbb{R}^n$. This is achieved by assigning to such a point cloud a parameterized sequence of nested shapes, called a *filtration*, and then studying how the topological features (connected components, holes, voids, and higher dimensional analogues) of these shapes vary as the parameter changes.

Definition 1: Let X be a topological space. A *single parameter filtration of* X is a collection of subspaces X_t of X for each $t \in \mathbb{R}$ such that $X_s \subset X_t$ whenever $s \leq t$.

An instructive example of a filtration is given by drawing a ball of radius t around each point in a point cloud, and letting these balls grow with the parameter t. We will refer to this as the *Čech filtration*. It is intuitive that new topological features (e.g. loops) will appear (*birth*) and be filled in (*death*) as t increases and the balls grow. To make this precise, we will make use of the mathematical theory of *homology*[2].

Homology (see (14) for a technical description) assigns to a given shape X a vector space[3] $H_k(X)$, a basis for which corresponds to the k-dimensional topological features in X. Furthermore, this assignment satisfies the *functoriality* property, a consequence of which is that an inclusion $X \subset Y$ induces a linear map $H_k(X) \to H_k(Y)$ which encodes how the topological features of X are realized in the larger space Y. The upshot of this is that one can associate to a filtration $(X_t)_{t\in\mathbb{R}}$ a sequence of vector spaces $(H_k(X_t))_{t\in\mathbb{R}}$ linked by linear maps $H_k(X_s) \to H_k(X_t)$ whenever $s < t$. This sequence contains precisely all the information of how the k-dimensional topological features of the filtration varies as the parameter changes.

Remarkably, the structure theorem of Zomorodian and Carlsson (15) guarantees that, under reasonable assumptions on the underlying filtration, all of this information can be completely summarized in a single object called a *barcode*. This barcode is a multiset of intervals, one for each topological feature in the filtration, whose endpoints indicate the birth and death parameters of the underlying feature.

A key strength of persistent homology is that it is robust to perturbations: slightly moving the points in the point cloud does not significantly alter the resulting barcode. However, single-parameter persistent homology is highly sensitive to outliers. For example, introducing a single point into the center of a ring of points will kill the resulting 1-dimensional feature, drastically altering the resulting barcode. This is problematic when there is a possibility of misclassifications, as is the case in this work.

B.2. Multiparameter persistence. To deal with outliers, we will introduce a second parameter which filters out points below a certain density. We thus need to generalize the single-parameter filtration introduced in Definition 1. We first endow $\mathbb{R}^n$ with the following poset structure which defines when $\mathbf{s} \leq \mathbf{t}$ for pairs $\mathbf{s}, \mathbf{t} \in \mathbb{R}^n$:

$$(s_1, \ldots, s_n) \leq (t_1, \ldots, t_n) \iff s_i \leq t_i \text{ for all } i \in \{1, \ldots, n\}. \quad \textbf{(16)}$$

Definition 2: Let X be a topological space. An *n-parameter filtration of* X is a collection of subspaces $X_{\mathbf{t}}$ of X for each $\mathbf{t} \in \mathbb{R}^n$ such that $X_{\mathbf{s}} \subset X_{\mathbf{t}}$ whenever $\mathbf{s} \leq \mathbf{t}$.

Note that when $n = 1$ we recover the single-parameter filtration of Definition 1.

A key example of a multiparameter filtration for $n = 2$ extends the single-parameter Čech filtration to depend on an extra filtering function. We will take this filtering function to be *codensity*.

[2]Not to be confused with the entirely distinct notion of homology in biology.
[3]Here we take homology with coefficients in $\mathbb{F}_2$

Definition 3: Suppose $P \subset \mathbb{R}^n$ is a finite point cloud. For $k \geq 1$, the *k-codensity* function $\rho_k \colon P \to \mathbb{R}$ is given by

$$\rho_k(p) = \frac{1}{k}\sum_{i=1}^{k} \|p - p_{(i)}\|$$

for each $p \in P$, where $p_{(i)}$ is the i-th nearest neighbor of p in P.

Definition 4: Suppose $P \subset \mathbb{R}^n$ is a finite point cloud with corresponding codensity $\rho_k \colon P \to \mathbb{R}$. For a given subset $Q \subset P$ write

$$B_r(Q) = \{x \in X : \|x - q\| \leq r \text{ for some } q \in Q\}$$

for the r-neighborhood about Q. For each $k \geq 1$, there is a 2-parameter filtration given by

$$X_{r,t} = B_r\left(\rho_k^{-1}((-\infty, t])\right).$$

We call these filtrations *Čech-codensity filtrations*.

Note that fixing the codensity parameter t and varying r is equivalent to taking the single-parameter Čech filtration over the filtered point cloud $\rho_k^{-1}((-\infty, t]))$. The idea is that at low values of t only the densest points are included, and so by increasing t it is possible to track the topological effect of including less dense points into the filtration. In practice, the Čech-codensity filtration is impractical to work with. In its place, it is typical to consider the *Rips-codensity* filtration, which can be seen as an approximation to the Čech-codensity filtration.

Just as before, by applying homology one arrives at a collection of vector spaces $(H_k(X_{\mathbf{t}}))_{\mathbf{t}\in\mathbb{R}^n}$ linked by linear maps describing how topological features are included from one set of parameter values to another. This is a special example of the following more general kind of algebraic object.

Definition 5: An *n-parameter multiparameter persistence module W* consists of the following data:

- A vector space $W_{\mathbf{t}}$ for each $\mathbf{t} \in \mathbb{R}^n$;
- A linear map $\iota_{\mathbf{s},\mathbf{t}} \colon W_{\mathbf{s}} \to W_{\mathbf{t}}$ for each pair $\mathbf{s}, \mathbf{t} \in \mathbb{R}^n$ whenever $\mathbf{s} \leq \mathbf{t}$.

In addition, the linear maps must satisfy:

- $\iota_{\mathbf{t},\mathbf{t}} = \mathbf{1}_{W_{\mathbf{t}}}$ for each $\mathbf{t} \in \mathbb{R}^n$
- $\iota_{\mathbf{s},\mathbf{t}} \circ \iota_{\mathbf{r},\mathbf{s}} = \iota_{\mathbf{r},\mathbf{t}}$ for each triple $\mathbf{r}, \mathbf{s}, \mathbf{t} \in \mathbb{R}^n$ whenever $\mathbf{r} \leq \mathbf{s} \leq \mathbf{t}$.

In other words, a multiparameter persistence module is precisely a functor $W \colon (\mathbb{R}^n, \leq) \to \mathrm{Vect}$.

In the single-parameter setting, the decomposition of these persistence modules gives rise to the barcode. However, in the multiparameter setting there is no such complete description of the information inside a persistence module. To arrive at an interpretable summary it is therefore necessary to define a representation that discards some of the included information.

B.3. MPH landscapes. For single-parameter persistent homology, Bubenik proposed persistence landscapes as a vectorization of the barcode (16). Later, Vipond generalized this notion to give the *multiparameter persistence landscape* (10), which is a vectorized invariant of multiparameter persistence modules. Vipond et al. (12) later applied this invariant to the study of immune cell spatial patterning in tumors, directly motivating the application of multiparameter persistence landscapes in this work.

Definition 6: Let W be an n-parameter multiparameter persistence module. The *(multiparameter) persistence landscape associated to W* is a function $\lambda \colon \mathbb{N} \times \mathbb{R}^n \to \mathbb{R}$ given by

$$\lambda(k, \mathbf{t}) = \sup\left\{\varepsilon > 0 : \beta_{\mathbf{t}-\varepsilon\mathbf{1}, \mathbf{t}+\varepsilon\mathbf{1}} > k\right\},$$

where $\beta_{\mathbf{s},\mathbf{t}} = \mathrm{rank}(\iota_{\mathbf{s},\mathbf{t}} \colon W_{\mathbf{s}} \to W_{\mathbf{t}})$ and we take the convention that the supremum of the empty set is 0. We will also write $\lambda_k \colon \mathbb{R}^n \to \mathbb{R}$ for the function $\lambda_k(\mathbf{t}) = \lambda(k, \mathbf{t})$.

If the persistence module is acquired by taking homology of a multiparameter filtration, one can think of $\lambda_k(\mathbf{t})$ as describing the 'significance' of the k-th most significant topological feature in the filtration at the parameter $\mathbf{t} \in \mathbb{R}^n$. In particular, λ_1 records the significance of the most significant feature.

A particularly useful property of persistence landscapes is that they can be averaged, which is notably not a property of barcodes: there exist simple examples of two barcodes with multiple Fréchet means. Given a family $\lambda^1, \ldots, \lambda^N$ of persistence landscapes,

their *average persistence landscape* $\overline{\lambda}$ is taken to be the pointwise average:

$$\overline{\lambda}_k(\mathbf{t}) = \frac{1}{N}\sum_{i=1}^{N} \lambda_k^i(\mathbf{t}). \quad \textbf{(17)}$$

Supplementary Methods 2: Data analysis

Here we describe in more detail the data analysis carried out in the main text. We begin by describing our implementation of the TopACT methodology for square grids. We then describe how TopACT and MPH landscapes are applied to clinical Stereo-seq and Xenium data.

A. TopACT implementation. We provide a Python package for TopACT with support for classification of 2D square grids as described in Algorithm 1. The package is modular and flexible, so it is possible for example to substitute in a custom local classifier in place of the provided SVM classifier.

A.1. Gene filtering. We performed minimal gene filtering in our experiments, making use of the ability of SVM classifiers to maintain performance with high dimensionality. For each spatial sample, we restrict to the set of genes present in both the sample and the snRNA-seq reference. If the user wishes to make use of a different local classifier, such as a neural network, it is likely that more gene filtering would be necessary.

A.2. Learning a local classifier. For our experiments, we make use of annotated snRNA-seq data as described in the main text. We first filter out all genes that do not appear in the spatial data under consideration. Let C be the resulting snRNA-seq count matrix, so that C_{ij} is the number of counts of gene j in sample i. The rows of the matrix C are normalized via the transformation

$$C'_{ij} = \log\left(\frac{10^5 C_{ij}}{\sum_{j'} C_{ij'}} + 1\right). \quad \textbf{(18)}$$

The columns of C' are then scaled to have unit variance, and the resulting feature matrix is used as training data for a linear SVM classifier. We use the SVM implementation in scikit-learn 1.1.1 (17) with default settings. The same normalisation pipeline is used when the local classifier is applied for spatial classifications.

A.3. Hyperparameters. TopACT requires two hyperparameters: a maximal radius $r_{\max}$ and a confidence threshold θ. For Stereo-seq data, we set the maximal radius $r_{\max}$ to be 9, which for the mouse kidney data approximates the radius of a single cell (9 spots $\approx 6.4\,\mu\text{m}$). We also set a minimum radius $r_1 = 3$ to improve efficiency. TopACT is implemented so that different values of the confidence threshold θ can be manually compared after classification is run, allowing for θ to be varied per experiment. We set $\theta = 0.5$ for synthetic data, $\theta = 0.7$ for mouse kidney Stereo-seq data, $\theta = 0.9$ for human kidney Xenium data, and $\theta = 0.5$ for mouse brain Stereo-seq data.

B. Stereo-seq data analysis. Here we describe how we ran TopACT on the mouse kidney Stereo-seq (7) data described in the main text. We analysed ten slices (4 from a control kidney, and 2 and 4 respectively from two treated kidneys).

B.1. Defining sample boundaries. For computational efficiency, we restricted cell type classifications to a convex hull approximating the underlying sample shape. In detail, we consider the gene density at each spot, i.e. the mean number of reads in a square of side length 21 centered on the spot. We then take the boundary of a sample to be the convex hull of all points with sufficiently high (> 5) density (see Supplementary Fig. 1). This ensures that computation time is not wasted on the boundary region of the sample, which we found had insufficient transcript counts to yield meaningful classifications.

B.2. TopACT output. We ran TopACT on each of the ten mouse kidney samples, restricted to the previously described regions. For the local classifier, we used an SVM classifier trained from snRNA-seq data annotated with three classes:

1. Podocyte cell,
2. Immune cell,
3. Other cell type.

B.3. Detecting single cells from TopACT output. We use a standard image analysis pipeline to extract single cell loci from TopACT output. In detail, for a given cell type and sample we produce a binary image representing spots that are classified with the given cell type. We then perform a difference of Gaussians (DoG) blob detection (19) (computed using scikit-image (20)) on a Gaussian smoothing of this binary image to extract single cell loci (see Supplementary Fig. 2A).

B.4. Validation via podocyte prediction. To validate the performance of TopACT on real-world data, we tested its ability to detect podocyte cells. Podocyte cells colocalise almost exclusively with glomeruli, which are large enough that they can be easily detected by existing methods at Bin 20 (i.e. with expression pooled into square bins with a side-length of 20 spots). This provides an ideal ground truth for validation. In detail, we used Seurat (21) with the same procedure as in the main text (*Single-nucleus clustering*) to produce a cell type annotation of each mouse sample at Bin 20 resolution. A pipeline similar to that used for single-cell detection was then used at Bin 20 resolution to detect regions of high podocyte density (Supplementary Fig. 2B). We took these regions to be ground truth glomeruli.

We then used the cell localisation pipeline to extract single podocyte cell loci from TopACT output. Supplementary Fig. 3 shows that these predicted podocyte cells strongly colocalise with the ground truth glomeruli, validating the use of TopACT on these data. Furthermore, violin plots show that TopACT-predicted podocyte cells are enriched in key podocyte marker genes (Supplementary Fig. 4.

B.5. Validation on tubule cells. For further validation we used TopACT to classify distal convoluted tubule (DCT) and proximal tubule (PT) cells in the same mouse kidney tissue. First, we compared locations of TopACT-predicted tubule cells with expression patterns of key marker genes, finding that TopACT predictions coincide with areas of high density of these markers (Figures 5 and 6). Moreover, violin plots of expression of these marker genes in both TopACT-predicted tubule cells, alongside TopACT-predicted podocyte cells and background cells sampled uniformly at random, show that TopACT predicted cells express their respective marker genes at a higher rate as expected, see Figure 4. This provides further validation of TopACT's ability to call cells in real world Stereo-seq data.

B.6. Generating patches. To normalize for area and to facilitate comparison between glomerular and non-glomerular regions, we split each kidney sample into square patches of side length 150 spots. Each of these patches is either glomerular (centered on a glomerulus) or non-glomerular (disjoint from any glomerular region). The glomerular patches were centered on the glomeruli locations extracted from Bin 20 data. To produce non-glomerular patches, we then randomly sampled non-overlapping patches to saturate the remaining area inside these boundaries. We repeated this process several times for each sample, and selected the resulting patch decomposition with the most coverage (i.e. the greatest number of patches). We then discarded glomerular patches with less than 90% overlap with the sample boundaries defined earlier and shown in Supplementary Fig. 1. This yielded 269 glomerular patches (108 control, 161 treated) and 310 non-glomerular patches (130 control, 180 treated) (see Extended Data Figure 4).

B.7. MPH landscapes. For each patch, we produced a point cloud where points correspond to spots classified as Immune by TopACT. For each of these point clouds, multiparameter persistence homology of the Rips-codensity filtration was computed with RIVET (23), from which MPH landscapes were computed using the code from (12). For the filtration, we used ρ_5 for codensity (see Definition 3) and set the maximum Rips radius to 100 spots. In RIVET we set the resolution parameter to 30. We then computed average multiparameter persistence landscapes (as in Eq. (17)) for control and treated glomerular patches.

Supplementary References

1. PL Ståhl, et al., Visualization and analysis of gene expression in tissue sections by spatial transcriptomics. *Science* **353**, 78–82 (2016).
2. SG Rodriques, et al., Slide-seq: A scalable technology for measuring genome-wide expression at high spatial resolution. *Science* **363**, 1463–1467 (2019).
3. RR Stickels, et al., Highly sensitive spatial transcriptomics at near-cellular resolution with Slide-seqV2. *Nat. Biotechnol.* **39**, 313–319 (2021).
4. BE Boser, IM Guyon, VN Vapnik, A Training Algorithm for Optimal Margin Classifiers in *Proceedings of the fifth annual workshop on Computational learning theory*. pp. 144–152 (1992).
5. C Cortes, V Vapnik, Support-Vector Networks. *Mach. Learn.* **20**, 273–297 (1995).
6. JC Platt, Probabilistic Outputs for Support Vector Machines and Comparisons to Regularized Likelihood Methods. *Adv. Large Margin Classif.* **10**, 61–74 (1999).
7. A Chen, et al., Spatiotemporal transcriptomic atlas of mouse organogenesis using DNA nanoball-patterned arrays. *Cell* **185**, 1777–1792.e21 (2022).
8. S Vickovic, et al., High-definition spatial transcriptomics for in situ tissue profiling. *Nat. Methods* **16**, 987–990 (2019).
9. CS Cho, et al., Microscopic examination of spatial transcriptome using Seq-Scope. *Cell* **184**, 3559–3572.e22 (2021).
10. O Vipond, Multiparameter Persistence Landscapes. *J. Mach. Learn. Res.* **21**, 1–38 (2020).
11. MB Botnan, M Lesnick, An Introduction to Multiparameter Persistence. *arXiv preprint arXiv:2203.14289* (2022).
12. O Vipond, et al., Multiparameter persistent homology landscapes identify immune cell spatial patterns in tumors. *Proc. Natl. Acad. Sci.* **118** (2021).
13. N Otter, MA Porter, U Tillmann, P Grindrod, HA Harrington, A roadmap for the computation of persistent homology. *EPJ Data Sci.* **6**, 1–38 (2017).
14. Allen Hatcher, *Algebraic Topology*. (Cambridge University Press), (2002).

15. A Zomorodian, G Carlsson, Computing Persistent Homology in *Proceedings of the Twentieth Annual Symposium on Computational Geometry*, SCG '04. p. 347–356 (2004).
16. P Bubenik, Statistical topological data analysis using persistence landscapes. *J. Mach. Learn. Res.* **16**, 77–102 (2015).
17. F Pedregosa, et al., Scikit-learn: Machine learning in Python. *J. Mach. Learn. Res.* **12**, 2825–2830 (2011).
18. A Zeisel, et al., Molecular Architecture of the Mouse Nervous System. *Cell* **174**, P999-1014.E22 (2018).
19. DG Lowe, Distinctive Image Features from Scale-Invariant Keypoints. *Int. J. Comput. Vis.* **60**, 91–110 (2004).
20. S van der Walt, et al., scikit-image: image processing in Python. *PeerJ* **2**, e453 (2014).
21. Y Hao, et al., Integrated analysis of multimodal single-cell data. *Cell* (2021).
22. Z Liu, et al., SONAR enables cell type deconvolution with spatially weighted Poisson-Gamma model for spatial transcriptomics. *Nat. Commun.* **14**, 4727 (2023).
23. The RIVET Developers, RIVET (2020).

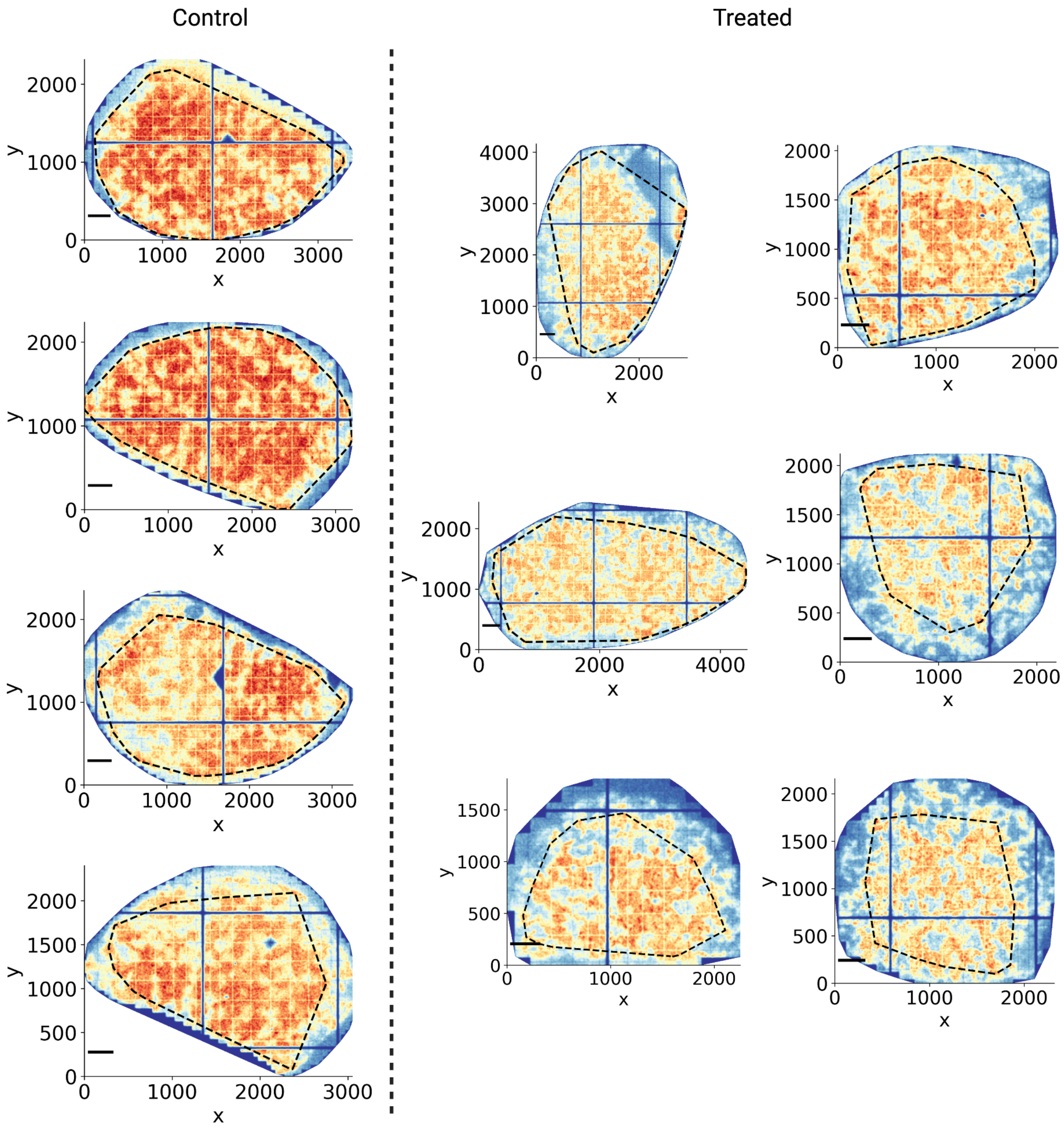

Supplementary Fig. 1 | Definition of sample boundaries based on transcript density. Each axis shows a single sample (Left: Control. Right: Treated). Black dashed lines show convex hulls of high-density regions in each sample, which are used to restrict later analysis. Background heatmaps show smoothed transcript count. Scale bars = 0.2mm.

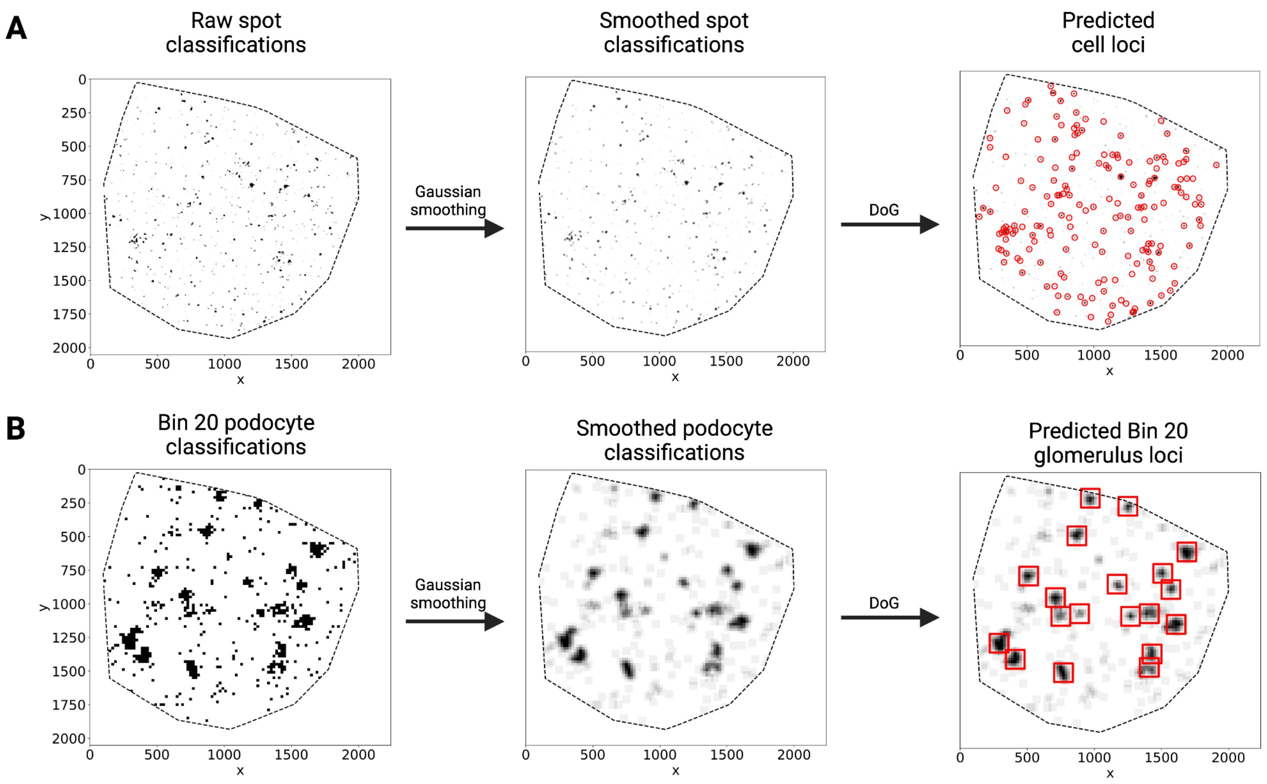

Supplementary Fig. 2 | Image analysis pipeline for detecting cells and glomeruli. (A) Extracting cell loci from spot-level cell type predictions. First, a binary image is produced indicating spots assigned the given cell type. Then, Gaussian smoothing is applied to produce a grayscale image. Finally, DoG blob detection (19, 20) is used to detect regions of high density of the given cell type. These regions are taken as predicted cell loci. In this example, immune cell loci are detected. **(B)** Extracting ground truth glomerulus loci from Bin 20 cell type predictions. The pipeline is identical to that set out in (A), but run specifically on podocyte predictions at Bin 20.

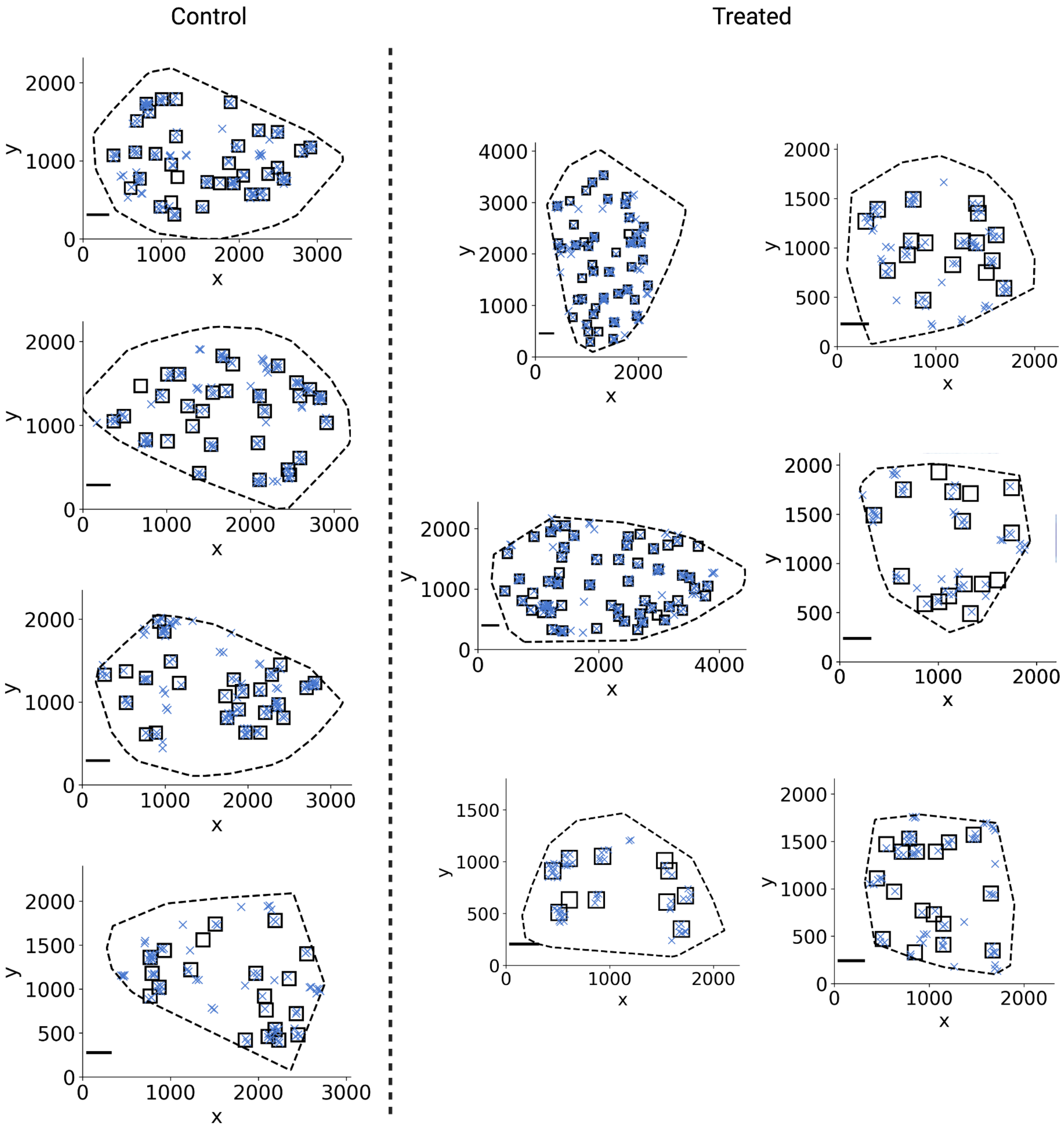

Supplementary Fig. 3 | Comparison of TopACT podocyte predictions to ground truth. TopACT predicted podocyte cells (blue cross) and ground truth glomeruli (black square) for each sample. Note that predicted podocytes colocalise with glomeruli, as expected, validating the use of TopACT on mouse kidney data. Dashed black lines show samples boundaries as in Supplementary Fig. 1. Scale bars = 0.2mm.

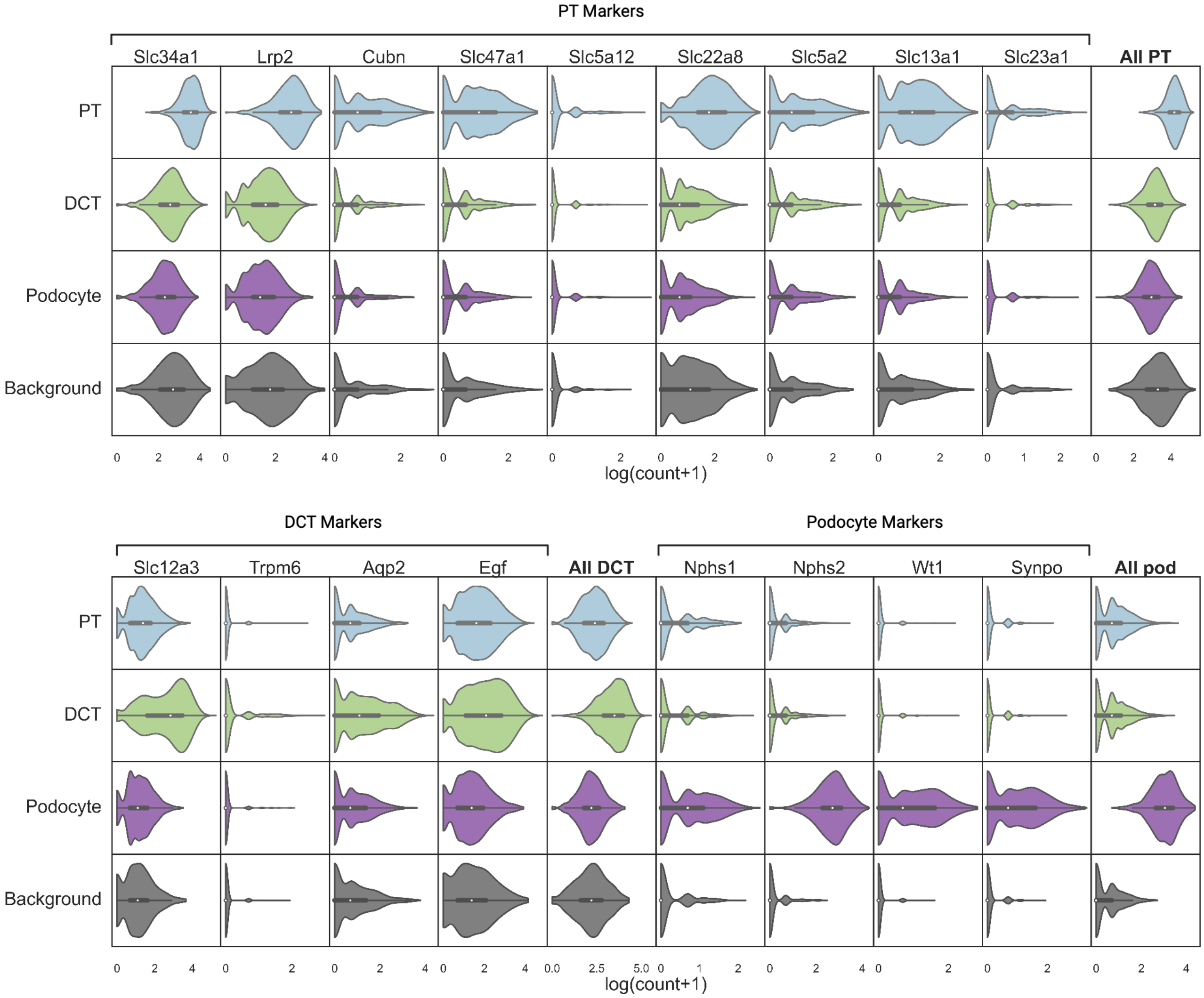

Supplementary Fig. 4 | Marker gene expression in predicted mouse kidney cells. Violin plots show expression of common markers of proximal tubule (PT) distal convoluted tubule (DCT), and podocyte cells, for TopACT predicted PT cells (blue), TopACT predicted DCT cells (green), TopACT predicted podocyte cells (purple), and randomly sampled background cells (grey), across all mouse kidney samples. Each plot corresponds to the expression counts of a single given marker gene in cells labelled with the given cell type across all samples. Top rows: PT markers. Bottom row, first half: DCT markers. Bottom row, second half: podocyte markers. Log scale.

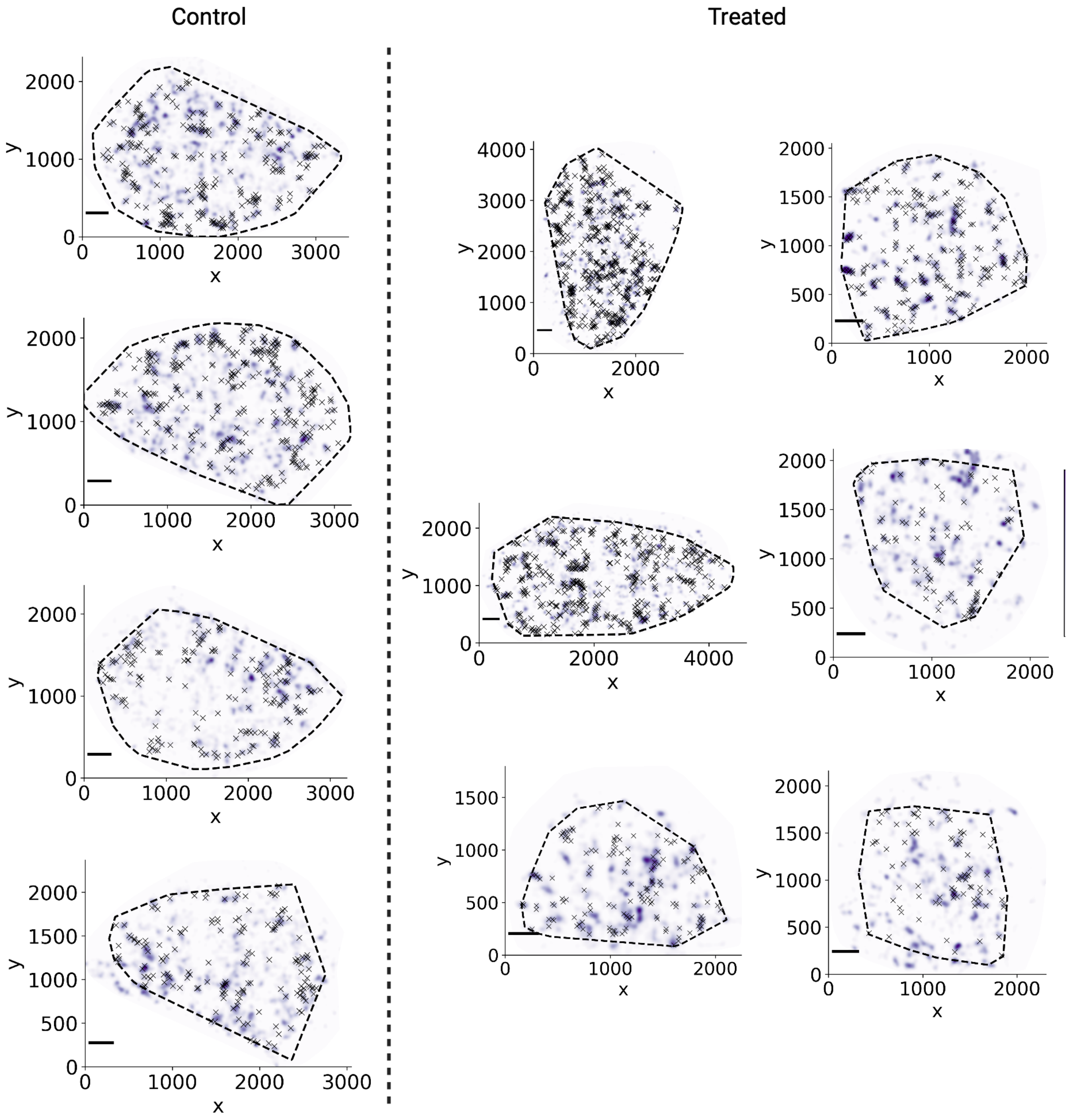

Supplementary Fig. 5 | Comparison of TopACT distal convoluted tubule (DCT) predictions to spatial distribution of marker genes. TopACT-predicted DCT cells (black cross) overlaid on map of combined density of DCT marker genes (blue background). Markers are *Slc12a3, Trpm6, Egf*, and *Aqp2*. Note that predicted DCT cells are found in areas of high marker gene expression as expected. Dashed black lines show samples boundaries as in Supplementary Fig. 1. Scale bars = 0.2mm.

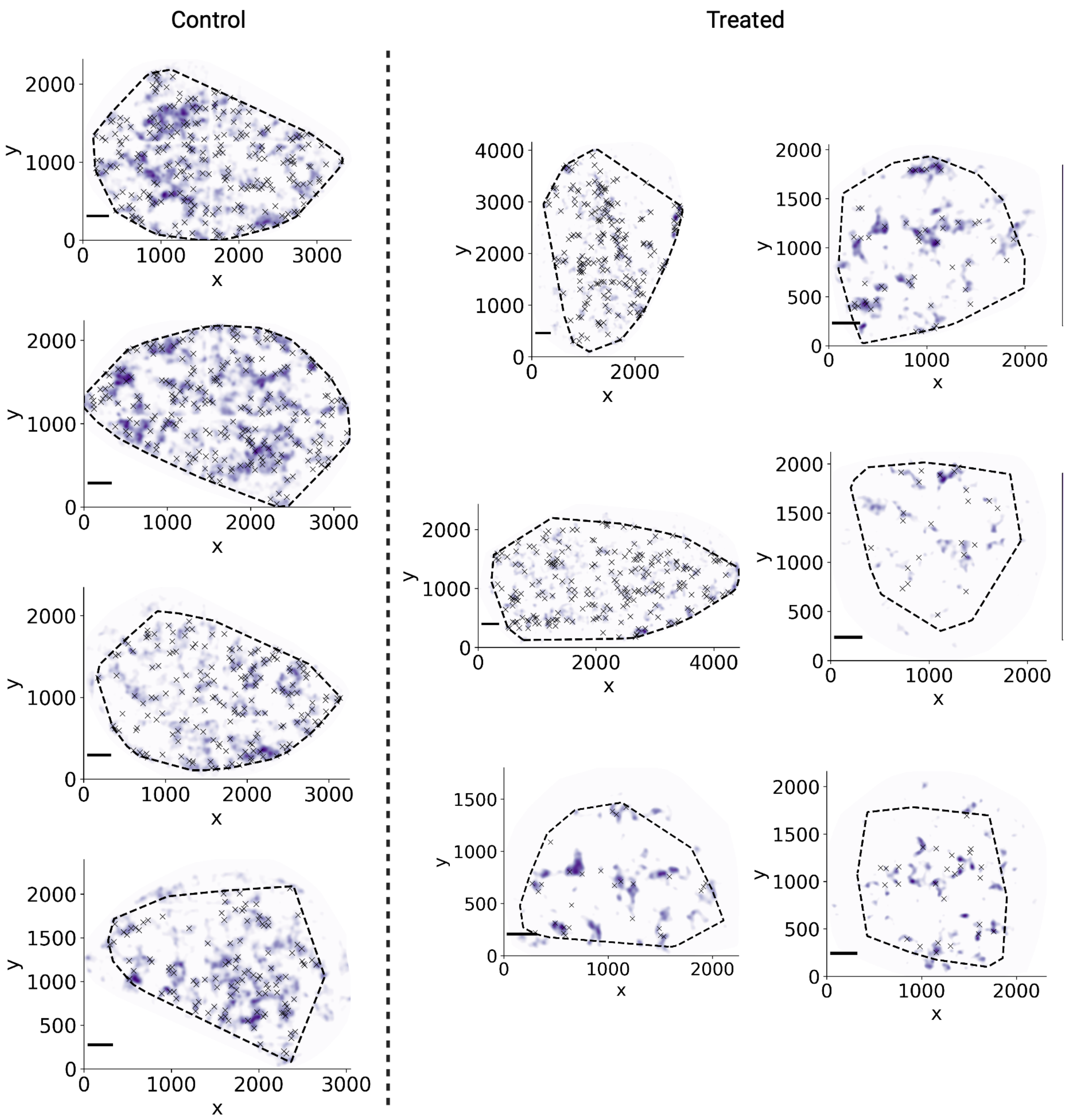

Supplementary Fig. 6 | Comparison of TopACT proximal tubule (PT) predictions to spatial distribution of marker genes. TopACT predicted PT cells (black cross) overlaid on map of combined density of PT marker genes (blue background). Markers are *Slc34a1, Lrp2, Cubn, Slc47a1, Slc5a12, Slc22a8, Slc5a2, Slc13a1,* and *Slc23a1*. Note that predicted PT cells are found in areas of high marker gene expression as expected. Dashed black lines show samples boundaries as in Supplementary Fig. 1. Scale bars = 0.2mm.
