# Supplementary material for: Multiscale topology classifies cells in subcellular spatial transcriptomics
Source: Nature. 2024 Jun 19;630(8018):943–9. doi: 10.1038/s41586-024-07563-1 (PMC11208150; doi:10.1038/s41586-024-07563-1)
Supplement: Supplementary file 2 — Reporting Summary [file 41586_2024_7563_MOESM2_ESM.pdf]

nature portfolio

Corresponding author(s): Katherine R. Bull, Heather A. Harrington

Last updated by author(s): Apr 22, 2024

Reporting Summary

Nature Portfolio wishes to improve the reproducibility of the work that we publish. This form provides structure for consistency and transparency in reporting. For further information on Nature Portfolio policies, see our Editorial Policies and the Editorial Policy Checklist.

Statistics

For all statistical analyses, confirm that the following items are present in the figure legend, table legend, main text, or Methods section.

n/a	Confirmed	
☐	☒	The exact sample size (*n*) for each experimental group/condition, given as a discrete number and unit of measurement
☐	☒	A statement on whether measurements were taken from distinct samples or whether the same sample was measured repeatedly
☐	☒	The statistical test(s) used AND whether they are one- or two-sided<br>*Only common tests should be described solely by name; describe more complex techniques in the Methods section.*
☒	☐	A description of all covariates tested
☒	☐	A description of any assumptions or corrections, such as tests of normality and adjustment for multiple comparisons
☐	☒	A full description of the statistical parameters including central tendency (e.g. means) or other basic estimates (e.g. regression coefficient) AND variation (e.g. standard deviation) or associated estimates of uncertainty (e.g. confidence intervals)
☐	☒	For null hypothesis testing, the test statistic (e.g. *F*, *t*, *r*) with confidence intervals, effect sizes, degrees of freedom and *P* value noted<br>*Give P values as exact values whenever suitable.*
☒	☐	For Bayesian analysis, information on the choice of priors and Markov chain Monte Carlo settings
☒	☐	For hierarchical and complex designs, identification of the appropriate level for tests and full reporting of outcomes
☒	☐	Estimates of effect sizes (e.g. Cohen's *d*, Pearson's *r*), indicating how they were calculated

Our web collection on statistics for biologists contains articles on many of the points above.

Software and code

Policy information about availability of computer code

Data collection	No software was used.
Data analysis	Standard software: Seurat 4.0 (snRNA-seq analysis); Zeiss Arivis 4.1.1(immunofluorescence image analysis); Scipy 1.8.1 and statannotations 0.5.0 (statistical tests). RIVET 1.0 (multiparameter persistent homology). spacexr 2.0 (RCTD implementation for cell type decomposition).<br><br>Custom software packages: TopACT 1.0 (spatial cell type classification). Available at https://gitlab.com/kfbenjamin/topact.<br><br>Custom Python 3.10 code for synthetic data experiments is available at https://github.com/katherine-benjamin/topact-paper. Code for experiments on clinical data will be made available in the same repository before publication.

For manuscripts utilizing custom algorithms or software that are central to the research but not yet described in published literature, software must be made available to editors and reviewers. We strongly encourage code deposition in a community repository (e.g. GitHub). See the Nature Portfolio guidelines for submitting code & software for further information.

Data

Policy information about availability of data

All manuscripts must include a data availability statement. This statement should provide the following information, where applicable:

- Accession codes, unique identifiers, or web links for publicly available datasets
- A description of any restrictions on data availability
- For clinical datasets or third party data, please ensure that the statement adheres to our policy

The immunofluorescence images are available via the Oxford University Research Archive at http://dx.doi.org/10.5287/ora-b7ob4g8ba. Raw Fastq files for Stereo-seq and snRNA-seq in murine lupus and control are available in the NCBI Sequencing Read Archive (SRA) with BioProject accession PRJNA1099448. The following data sets are deposited in the NCBI gene expression omnibus (GEO): the Seurat object from the murine snRNA-seq with accession GSE265819, the bin 1 matrix generated from the Stereo-seq dataset with accession GSE264321, raw Fastq and Seurat object for human kidney healthy snRNA-seq with accession GSE264393, Xenium data from human IgAN kidney with accession GSE264334. Mouse brain spatial data was obtained from https://db.cngb.org/stomics/mosta/ and corresponding brain scRNA-seq from the NCBI SRA with accession SRP135960. Output files for TopACT and related analysis are available at https://doi.org/10.5281/zenodo.10950538. Source data for figures are provided with this paper.

Research involving human participants, their data, or biological material

Policy information about studies with human participants or human data. See also policy information about sex, gender (identity/presentation), and sexual orientation and race, ethnicity and racism.

Reporting on sex and gender

The human samples included in this study are summarised below, mean sample age was 56, MEST score reflects IgAN disease classification (Trimarchi et al Kidney International 2017 PMID: 28341274):

Experiment Sample Name Sample ID Genotype Sex MEST-C Score
snRNA-Seq CTL#1 A1 CTL Female N/A
snRNA-Seq CTL#2 C3 CTL Male N/A
snRNA-Seq CTL#3 C4 CTL Female N/A
snRNA-Seq CTL#4 C5 CTL Female N/A
Xenium IgAN1 IgAN1 Disease Male M0E0S1T1C0

Reporting on race, ethnicity, or other socially relevant groupings

Self reported ethnicity data from the NHS record was collected, 3 White British, 2 Other.

Population characteristics

Age, gender, disease status, ethnicity and IgAN MEST score are provided above. Some data is pooled to ensure anonymity.

Recruitment

The IgAN sample analysed by Xenium was obtained via the Oxford Centre for Histopathology Research, from tissue obtained at the time of a clinically indicated diagnostic renal biopsy. the human healthy kidney samples were obtained via the Oxford Transplant Biobank from healthy kidney donors, who consented to provide a biopsy sample for research at the time of kidney donation.

Ethics oversight

The IgAN sample was obtained as part of an approved project (ORB/23/A064) within the Oxford Centre for Histopathology Research, approved under the Oxford Radcliffe Biobank tissue bank ethics, reference 19/SC/0173.
Healthy samples for snRNA-seq were obtained via an approved project (OTB006) as part of the Oxford Transplant Biobank, reference 19/SC/0529. Both studies were approved by the South Central -Oxford C Research Ethics Committee.

Note that full information on the approval of the study protocol must also be provided in the manuscript.

Field-specific reporting

Please select the one below that is the best fit for your research. If you are not sure, read the appropriate sections before making your selection.

☒ Life sciences ☐ Behavioural & social sciences ☐ Ecological, evolutionary & environmental sciences

For a reference copy of the document with all sections, see nature.com/documents/nr-reporting-summary-flat.pdf

Life sciences study design

All studies must disclose on these points even when the disclosure is negative.

Sample size

Due to the costly and complex processed involved in new ST techniques, small numbers of samples were used in these experiments. Importantly the focus of our manuscript is in developing and showcasing the method, here we are able to test TopACT across 4 different datasets (synthetic, murine kidney, murine brain and human kidney). Where comparison is made between disease and control for the murine lupus study, the TopACT findings are supported by analysis of 30 glomeruli per mouse in multiplex immunofluorescence performed in 3 treated and 3 control kidney mice. This 3 x 3 experiment is sufficiently powered at 80%, to detect a difference between mean intensities of 33% with a SD of 14%, with a 0.05 chance of a type I error, consistent with the variability of the multiplex imaging data (See Festing and

	Altman ILAR Journal 2002 PMID: 11506638).
Data exclusions	Experimental animals or human samples were not excluded from analysis
Replication	Spatial and snRNA sequencing experiments were not repeated due to the costly nature of these experiments, and the focus in the manuscript on methodological development
Randomization	Age matched Balb/C mice were allocated randomly to Imiquimod treatment or control. Cages were not mixed due to the risk of control animal exposure via grooming.
Blinding	Investigators were not blinded for snRNA and spatial, but the analysis pipelines were applied identically to all datasets. IF glomerular intensity was measured by an operator blinded to the treatment group.<br>For new human data, control samples are used to generate kidney snRNA seq data for supervised clustering and are not used to directly compare IgAN cell pathology with control. Given this, and the small sample numbers, adjustment for covariates is not relevant to the human data presented. However the sex, age, and eGFR are provided in this reporting summary for information.

Reporting for specific materials, systems and methods

We require information from authors about some types of materials, experimental systems and methods used in many studies. Here, indicate whether each material, system or method listed is relevant to your study. If you are not sure if a list item applies to your research, read the appropriate section before selecting a response.

Materials & experimental systems

n/a	Involved in the study
☐	☒ Antibodies
☒	☐ Eukaryotic cell lines
☒	☐ Palaeontology and archaeology
☐	☒ Animals and other organisms
☒	☐ Clinical data
☒	☐ Dual use research of concern
☒	☐ Plants

Methods

n/a	Involved in the study
☒	☐ ChIP-seq
☒	☐ Flow cytometry
☒	☐ MRI-based neuroimaging

Antibodies

Antibodies used	1. Ly6G/Ly6C (Gr-1) 1:400 (MAB1037-SP; R&D Systems)-Opal 480 1:150<br>2. CD4 1:500 (ab183685; Abcam)-Opal-520 1:150<br>3. CD8 1:800 (98941; Cell Signalling)-Opal 520 1:150<br>4. CD68 1:1200 (ab125212; Abcam)-Opal 570 1:150<br>5. CD19 1:600 (90176; Cell Signalling)-Opal 620 1:150<br>6. CD11b 1:80,000 (ab133357; Abcam)-Opal 690 1:150<br>7. E-cadherin 1:500 (3195; Cell Signalling)-Opal 780 1:25
Validation	Antibodies were validated by the manufacturersAl<br>Ly6G/Ly6C (Gr-1) (MAB1037-SP) RnD systems provide images showing efficacy for immunofluoresence (IF) in mouse splenocytes (https://www.rndsystems.com/products/mouse-ly-6g-ly-6c-gr-1-antibody-rb6-8c5_mab1037#product-details), see also Hayashida et al Blood 2009 PMID: 19638625<br>CD4 (ab183685) has been tested by abcam for Immunohistochemistry (IHC) on fresh or paraffin embedded tissue in mouse. See also Sui et al Nat Comms 2022 PMID: 36575174 for use in IF<br>CD8 (98941) Cell Signalling provide data showing use for IHC in mouse lung and spleen. See Wang et al Nat Comms 2018 PMID: 29422647 for use in IF in mouse.<br>CD68 (ab125212) Abcam demonstrate use in mouse for IHC, use for murine IF demonstrated e.g. in Golden et al PLoS Pathogens 2022 PMID: 35587473<br>CD19 (90176) Cell signalling provide data showing use in IHC in mouse. See Patel et al Sci Adv 2022 PMID: 35749506 for application in mouse IF.<br>CD11b (ab133357) abcam demonstrate use in mouse for IHC.<br>E-cadherin (3195) Cell signalling report mouse reactivity and show immunofluoresence in cell lines. IF in mouse embryos is shown in Alharatani et al Hum Mol Genet 2020 PMID: 32196547

Animals and other research organisms

Policy information about studies involving animals; ARRIVE guidelines recommended for reporting animal research, and Sex and Gender in Research

Laboratory animals	Female BALB/cOlaHsd mice, 5 weeks of age. Animals were housed in individually ventilated cages under specific pathogen free conditions at temperatures between 20–24°C and humidity 45-65%.
Wild animals	The study did not involve wild animals

Reporting on sex	Female mice were used as there is a sex bias in response to Imiquimod with females exhibiting a more severe phenotype. This mirrors the higher incidence of human lupus in females.
Field-collected samples	The study did not involve samples collected from the field.
Ethics oversight	All animal experiments were performed in accordance with the Animals (Scientific Procedures) Act 1986, amended 2012, with procedures reviewed by the clinical medicine Animal Care and Ethical Review Body and conducted under home office license P84582234, held by Katherine Bull.

Note that full information on the approval of the study protocol must also be provided in the manuscript.

Plants

Seed stocks	*Report on the source of all seed stocks or other plant material used. If applicable, state the seed stock centre and catalogue number. If plant specimens were collected from the field, describe the collection location, date and sampling procedures.*
Novel plant genotypes	*Describe the methods by which all novel plant genotypes were produced. This includes those generated by transgenic approaches, gene editing, chemical/radiation-based mutagenesis and hybridization. For transgenic lines, describe the transformation method, the number of independent lines analyzed and the generation upon which experiments were performed. For gene-edited lines, describe the editor used, the endogenous sequence targeted for editing, the targeting guide RNA sequence (if applicable) and how the editor was applied.*
Authentication	*Describe any authentication procedures for each seed stock used or novel genotype generated. Describe any experiments used to assess the effect of a mutation and, where applicable, how potential secondary effects (e.g. second site T-DNA insertions, mosiacism, off-target gene editing) were examined.*
